# Pheno-morphological and biochemical characterization of root nodules and associated root nodulating bacteria from *Pongamia pinnata* (L.) Pierre in the arid regions of India

**DOI:** 10.3389/fpls.2025.1717750

**Published:** 2025-12-18

**Authors:** Vipula Vyas, Sangeeta Singh, Sunil Choudhary, Tanmaya Kumar Bhoi, Prithwiraj Dey, Anuj Saraswat

**Affiliations:** 1 Forest Protection Division, Indian Council of Forestry Research and Education (ICFRE)-Arid Forest Research Institute, Jodhpur, Rajasthan, India; 2 Department of Botany, Shri Baldev Ram Mirdha Government College, Nagaur, Rajasthan, India; 3 Agricultural and Food Engineering Department, Indian Institute of Technology Kharagpur, West Bengal, India; 4 Department of Biology, United Arab Emirates University, Al Ain, United Arab Emirates

**Keywords:** *Pongamia pinnata*, root nodulating bacteria, nitrogen fixation, nodule morphology, antioxidant activity

## Abstract

**Introduction:**

*Pongamia pinnata* (L.) Pierre is a resilient leguminous tree valued for its biofuel potential and ability to flourish in marginal soils due to symbiotic nitrogen fixation by root-nodulating bacteria (RNB). Understanding the phenomorphological, soil, and biochemical characteristics of its associated RNB is essential for enhancing productivity in arid regions. This study aimed to characterize RNB isolates associated with *P. pinnata* and assess how soil properties and nodule biochemistry influence plant growth in the arid ecosystems of western Rajasthan.

**Methods:**

Twenty RNB isolates (PP-01 to PP-20) were collected from *P. pinnata* nodules across arid sites. Rhizospheric soil samples were analysed for physico-chemical parameters, including pH, EC, organic carbon, and nutrient contents. Nodules were examined for morphology and nitrogen-fixing activity. Biochemical profiling of isolates included phenolics, tannins, FRAP, and total antioxidant capacity. Seedling growth responses to individual isolates were evaluated under controlled conditions. Statistical analyses included multiple regression, stepwise regression, PCA, and hierarchical cluster analysis.

**Results:**

Soils were alkaline (pH 8.2–9.1) with moderate EC (1.18–1.89 dS m^-^¹) and heterogeneous nutrient availability. Nodules exhibited diverse morphology with active nitrogen fixation. Seedling growth differed significantly among isolates, with PP-18, PP-19, and PP-20 showing the highest performance. Biochemical traits varied widely; isolates PP-08, PP-09, PP-14, and PP-20 demonstrated superior antioxidant activity. Multiple regression identified nitrogen, potassium, pH, organic carbon, tannin, and antioxidant content as positive contributors to growth, while phosphorus, phenol, and EC were negative predictors (R² = 0.85). Stepwise regression indicated nitrogen, pH, organic carbon, and tannin as the most influential variables (R² = 0.61). PCA explained 98.8% of the total variance and distinctly separated isolates based on biochemical and growth characteristics. Cluster analysis grouped the twenty sites into three clusters corresponding to soil fertility gradients.

**Discussion:**

The study demonstrates that both soil nutrient status and nodule biochemical composition jointly regulate *P. pinnata* growth under arid conditions. High-performing isolates, particularly PP-18, PP-19, and PP-20, possess favourable physiological and biochemical attributes supportive of plant growth. The strong discriminatory power of PCA and clustering highlights the ecological differentiation among isolates across fertility gradients. These results underscore the potential of selecting site-specific, elite RNB strains to enhance *P. pinnata* productivity, soil fertility, and sustainable agroforestry in arid landscapes.

## Introduction

Nitrogen is one of the most critical macronutrients required by plants, forming the basis of proteins, nucleic acids, and chlorophyll (Yeremko et al., 2025). However, atmospheric nitrogen (N_2_), which constitutes nearly 78% of the Earth’s atmosphere, is inert and unavailable for direct plant uptake (Giordano et al., 2021). The process of biological nitrogen fixation (BNF), mediated by specialized prokaryotes such as Rhizobium spp (Mukherjee and Sen, 2021; Mrunalini et al., 2022), plays a pivotal role in converting atmospheric nitrogen into ammonia, which plants can utilize (Khare et al., 2025). In legumes, this symbiotic interaction is facilitated through the development of root nodules, specialized organs that house nitrogen-fixing bacteria (Nag et al., 2020). The establishment of nodulation involves intricate molecular signaling between the host plant and bacteria, including the secretion of flavonoids by the plant that activate bacterial nod genes and trigger the synthesis of Nod factors (Patil et al., 2024). These signaling compounds induce root hair curling, cortical cell division, and the formation of nodules. Within these nodules, differentiated Rhizobium cells (bacteroids) (Goyal et al., 2021) fix nitrogen using the enzyme nitrogenase, while leghemoglobin regulates oxygen supply, ensuring efficient nitrogen fixation. Effective nodules typically appear pink to reddish due to leghemoglobin, whereas ineffective nodules remain green or white (Villar et al., 2021), lacking nitrogen-fixing activity (Soumare et al., 2020; Khare et al., 2025).

*Pongamia pinnata* (syn. *Millettia pinnata*), commonly known as Karanj or Indian Beech, is a leguminous tree of immense economic, ecological, and medicinal significance (Ramanjaneyulu et al., 2025). Traditionally, it has been used for oilseed extraction, timber, fodder, shade, green manure, and medicine (Singh et al., 2021). Recently, *P. pinnata* has emerged as a promising biofuel crop owing to the high oil content of its seeds, providing a sustainable alternative to fossil fuels (Kumar N. et al., 2025). Its adaptability to degraded lands, tolerance to salinity and drought, and ability to establish symbiotic associations with nitrogen-fixing bacteria make it highly suitable for cultivation in marginal ecosystems (Nemenzo, 2016). Besides its economic importance, *P. pinnata* contributes to ecological sustainability by enhancing soil fertility through biological nitrogen fixation, thereby reducing dependence on synthetic fertilizers (Bhoi et al., 2024; Mehata et al., 2023; Sharma et al., 2024). This is particularly relevant for arid and semi-arid regions where soils are inherently poor in nutrients.

The arid regions of western Rajasthan, a representative dryland ecosystem of India, pose severe challenges for agriculture and forestry due to harsh climatic conditions, extreme temperatures, and erratic rainfall (Vyas et al., 2024; Singh and Sharma, 2025). The soils are generally sandy, deficient in organic carbon, and often saline-alkaline in nature, with low nitrogen, phosphorus, and potassium levels (Kundu et al., 2024; Naorem et al., 2023; Sharma et al., 2021). These conditions make it difficult for most conventional crops to survive, leading to reduced agricultural productivity. In such ecosystems, hardy perennial trees like *P. pinnata* provide a sustainable option (Saritha et al., 2025), as they can withstand abiotic stresses and simultaneously improve soil fertility through symbiotic nitrogen fixation. However, the success of nodulation and nitrogen fixation in such stressed environments largely depends on the compatibility between host plants and the indigenous Rhizobium strains present in the soil (Goyal and Habtewold, 2023).

Despite the importance of *P. pinnata*, relatively little is known about the diversity and efficiency of its root nodule symbiosis in arid ecosystems (Bhati et al., 2017). Most studies on *P. pinnata* have focused on its biofuel potential, oil yield, or agronomic management, with limited attention given to its microbial associations (Mulla and Shah, 2025). Moreover, the influence of soil parameters such as nutrient status, pH, electrical conductivity, and organic carbon content on nodule formation and function has not been systematically explored. Given that arid soils often harbor stress-adapted microbial populations, exploring the characteristics of *Rhizobium* spp. associated with *P. pinnata* in these regions is essential (Singh et al., 2023; Aguilar et al., 2025). Morphological features of nodules, such as size, shape, and color, provide important indicators of symbiotic performance, while biochemical traits such as leghemoglobin content and nitrogenase activity reveal their functional efficiency (Aguilar et al., 2025; Singh S. et al., 2025). Similarly, phenotypic and biochemical characterization of associated *Rhizobium* strains can help identify stress-tolerant and highly efficient inoculants that can be harnessed for sustainable agroforestry systems (Tilgam et al., 2022).

The characterization of nodules and associated *Rhizobium* spp. has broader implications for ecological restoration, biofertilizer development, and sustainable land management (Gebreegziabher et al., 2025). By identifying efficient strains capable of surviving in nutrient-deficient, saline-alkaline soils, it becomes possible to enhance nodulation and nitrogen fixation in *P. pinnata* plantations (Thakur et al., 2025). Such findings are directly relevant for developing location-specific bio-inoculants tailored to arid regions, thereby reducing dependency on chemical fertilizers and promoting soil health.

Considering these gaps, the present study focuses on the pheno-morphological and biochemical characterization of root nodules and associated *Rhizobium* spp. from *P. pinnata* growing in the arid regions of western Rajasthan. The main objectives are to collect rhizospheric soil samples from diverse arid locations, establish controlled pot experiments to evaluate plant growth and nodulation responses to different soils, characterize nodules morphologically in terms of size, shape, and color to assess their effectiveness, and conduct biochemical analyses of nodules and *Rhizobium* strains to determine their nitrogen-fixing potential and stress adaptability. The underlying hypothesis of this research is that arid soils of Rajasthan harbor distinct and stress-adapted *Rhizobium* populations, which vary in their ability to form effective nodules with *P. pinnata*, and that some of these strains can be exploited as region-specific bio-inoculants. By identifying and characterizing effective nodules and associated microbial strains, this work will contribute to the development of biofertilizers suitable for arid and semi-arid ecosystems. Such biofertilizers can enhance the productivity of *P. pinnata* plantations, improve soil fertility, and reduce the reliance on synthetic nitrogen inputs. Furthermore, large-scale plantations of *P. pinnata* supported by efficient nitrogen-fixing symbioses can serve as a sustainable strategy for biofuel production, rural livelihood enhancement, and ecological restoration in fragile dryland ecosystems (Singh S. et al., 2025).

## Materials and methods

### Survey and sampling

A comprehensive survey was carried out across diverse geographical locations in western Rajasthan to collect rhizospheric soil samples associated with *P. pinnata* (**Table 1**). A total of 8 sites were strategically selected to represent a wide range of soil types, climatic conditions, and ecological diversity, ensuring that the study captured variability in edaphic and environmental factors influencing rhizosphere microbial communities. At each site, healthy *P. pinnata* trees were carefully identified, and rhizospheric soil was collected from the root zone at a depth of 10–20 cm using sterile tools to avoid contamination. Approximately 4 kg of soil per tree was collected, a quantity chosen based on previous studies (Valiullin et al., 2024) demonstrating that this amount provides sufficient material for both physicochemical analyses and isolation of microbial communities while minimizing disturbance to the tree root system. The soil was collected in sterile polyethylene bags and transported to the laboratory under cooled conditions to preserve microbial viability. In the laboratory, the soil samples were thoroughly homogenized, passed through a 2 mm sieve to remove stones and debris, and stored at 4°C until further analyses, ensuring that both the physical and biological integrity of the samples was maintained for subsequent studies.

**Table 1:** Geographical locations, coordinates, and isolate codes of *Pongamia pinnata* rhizobial isolates collected from western Rajasthan.

S. No.	District	Site/location	Site code	Latitude (N)	Longitude (E)	Isolate code
1	Barmer	Pachpadra	S1	25°55′19.1″ N	72°15′34.1″ E	PP-01
		Sindhari	S2	25°34′15.3″ N	71°54′32.3″ E	PP-02
2	Churu	Randhisar	S3	27°53′58.5″ N	74°31′19.3″ E	PP-03
		Sardarsahar	S4	27°48′38.6″ N	74°26′04.7″ E	PP-04
		Tal Chappar	S5	28°27′22.0″ N	74°32′21.9″ E	PP-05
3	Jodhpur	AFRI Nursery	S6	26°14′02.4″ N	73°01′12.0″ E	PP-06
		Mogra Khurd	S7	26°07′30.2″ N	73°03′40.1″ E	PP-07
		Balesar	S8	26°27′41.3″ N	73°15′12.6″ E	PP-08
		Mathania	S9	26°23′20.8″ N	73°28′18.5″ E	PP-09
4	Jhunjhunu	Jhunjhunu	S10	28°02′28.6″ N	75°29′39.5″ E	PP-10
		Pilani	S11	28°22′02.6″ N	75°35′07.4″ E	PP-11
		Udaipurwati	S12	27°43′32.4″ N	75°29′07.7″ E	PP-12
		Bissau	S13	27°59′05.7″ N	75°36′12.2″ E	PP-13
5	Sikar	Chala	S14	27°40′15.9″ N	75°40′29.6″ E	PP-14
		Harsh	S15	27°33′40.7″ N	75°09′33.7″ E	PP-15
		Losal	S16	27°23′58.2″ N	74°51′00.9″ E	PP-16
		Fatehpur	S17	27°59′42.8″ N	74°57′55.3″ E	PP-17
		Laxmangarh	S18	27°49′10.1″ N	75°02′18.6″ E	PP-18
		Danta Ramgarh	S19	27°27′45.2″ N	75°29′11.4″ E	PP-19
		Reengus	S20	27°21′51.4″ N	75°34′44.9″ E	PP-20

### Soil nutritional analysis

The collected rhizospheric soil samples were analyzed in triplicate to assess their nutrient status, with a primary focus on macronutrients, namely nitrogen (N), phosphorus (P), and potassium (K). Triplicate analyses were performed as technical replicates from the same homogenized soil sample to ensure analytical precision, and certified reference materials and procedural blanks were included to validate the accuracy and reliability of the methods. Total nitrogen content was determined using the Kjeldahl method (Goyal et al., 2022), which involved digestion of the soil samples with concentrated sulfuric acid, followed by distillation and titration with a standard acid to quantify ammonium nitrogen. Available phosphorus was measured using the Olsen’s method, wherein soil samples were extracted with 0.5 M NaHCO_3_ at pH 8.5, and the phosphorus content was subsequently quantified spectrophotometrically (Denovix; DS-11+) using the molybdenum blue method at 882 nm (Ameer et al., 2024). Exchangeable potassium was estimated using flame photometry (Elico, C-378); soil samples were extracted with 1 N ammonium acetate solution at pH 7.0, and the potassium concentration was measured to evaluate soil fertility (Ullah et al., 2022). In addition to these macronutrients, other critical soil properties, including pH, electrical conductivity (EC), organic carbon content, and soil texture which was determined using the feel method, were also recorded in triplicate to ensure accuracy and reproducibility (Singh G. et al., 2025).

### Trapping experiment

To evaluate the effect of rhizospheric soil from different locations on the growth of *P. pinnata*, a controlled pot experiment was established, incorporating a trapping experiment to assess nodule formation (Mendoza-Suárez et al., 2021). Healthy *P. pinnata* seeds were surface-sterilized with 0.1% mercuric chloride for 2–3 minutes and thoroughly rinsed with sterile distilled water to remove surface contaminants. The seeds were then pre-soaked in sterile water for 24 hours to enhance germination. Sterile plastic pots (30 cm diameter) were filled with 3 kg of rhizospheric soil collected from each site, and three seeds were sown per pot. After germination, seedlings were thinned to one per pot to avoid competition for nutrients, water, and space. The trapping experiment was conducted in triplicate for each soil sample, resulting in three independent replicates per site to ensure reproducibility and statistical reliability of the results. All pots were arranged in a completely randomized design (CRD) under natural environmental conditions, with regular irrigation using distilled water to maintain optimum soil moisture. Plants were grown for 90 days, and growth parameters, including plant height, number of leaves, stem diameter, and root length, were recorded at regular intervals to evaluate the influence of soil origin on seedling development and vigor. Upon reaching a predetermined growth stage, the plants were carefully uprooted to avoid damage to the root system, and the nodules were separated from the roots for subsequent morphological, biochemical, and microbial characterization.

### Nodule characterization

#### Morphological characterization

The nodules formed on the roots of *P. pinnata* were carefully separated and subjected to detailed morphological characterization to assess their structure and potential effectiveness. According to Paudyal et al. (2021), morphological observations were conducted in triplicate for each soil treatment, with three nodules analyzed per replicate to ensure accuracy and reproducibility of the data. For each site, nodules were collected from 3–5 healthy plants, and nodules from individual plants were assessed separately rather than pooled, allowing for plant-specific comparisons and evaluation of variability within a site. Nodule size was measured using a Vernier caliper, recording the length and diameter of each nodule to determine variation across different soil treatments. Nodules were classified based on their shape into categories such as spherical, elongated, or irregular, allowing for comparative analysis of nodule development under varying soil conditions. Additionally, both external and internal colors of the nodules were recorded, as the coloration is indicative of nodule functionality: effective nodules were identified by a pink to reddish hue, reflecting the presence of leghemoglobin, whereas ineffective nodules appeared green or white. These morphological observations, carried out systematically across all replicates, provided essential baseline information on the health, development, and symbiotic potential of nodules across different rhizospheric soils, forming a critical component of the nodule characterization study.

#### Biochemical characterization

Root nodules from 20 P*. pinnata* isolates (PP-01 to PP-20) were collected, washed thoroughly to remove adhering soil, blotted dry, and immediately frozen in liquid nitrogen. For each isolate, three biological replicates (2.0 g fresh weight each) were prepared, and the frozen nodules were ground to a fine powder using a pre-chilled mortar and pestle. The powdered tissue was homogenized in 10 ml of ice-cold phosphate buffer (50 mM, pH 7.8) and centrifuged at 12,000 rpm for 20 min at 4°C. The resulting supernatant was collected in 2.5 ml microtubes and stored at −20°C until further use. Biochemical assays were performed with three technical replicates for each biological replicate. Total phenolic content was determined following Singleton and Rossi (1965) by mixing 200 µl of the extract with 300 µl of distilled water and 0.1 ml of Folin–Ciocalteu reagent, incubating for 15 min in the dark, adding 2.5 ml of saturated sodium carbonate, and incubating further for 30 min at room temperature before recording absorbance at 760 nm using an ELISA plate reader. A reagent blank containing distilled water instead of extract and a sample blank (extract without Folin–Ciocalteu reagent) were included to correct background absorbance. A gallic acid standard curve (0–200 µg ml^-^¹) was used for quantification, and results were expressed as mg gallic acid equivalent (GAE) g^-^¹ FW. Total tannins were quantified following Amorim et al. (2008) by adding 0.1 ml of extract to a volumetric flask containing 7.5 ml of distilled water, 0.5 ml of Folin–Ciocalteu reagent, and 1.0 ml of 35% sodium carbonate, making up the volume to 10 ml with distilled water, mixing thoroughly, incubating for 30 min at room temperature, and recording absorbance at 700 nm. Similar reagent and sample blanks were prepared, and gallic acid standards (0–200 µg ml^-^¹) were used to generate the calibration curve, with results expressed as mg GAE g^-^¹ FW. Ferric reducing antioxidant power (FRAP) was assayed according to Benzie and Strain (1999) by adding 3.8 ml of freshly prepared FRAP reagent to 100 µl of extract, incubating at room temperature for 30 min in the dark, and measuring absorbance at 593 nm. A reagent blank containing FRAP reagent without extract was included to zero the spectrophotometer, and a calibration curve was prepared using ascorbic acid (0–500 µg ml^-^¹) to express the reducing power as µmol Fe²^+^ g^-^¹ FW. Total antioxidant activity was estimated by the phosphomolybdenum method (Prieto et al., 1999), wherein 100 µl of the extract was mixed with 200 µl of distilled water and 3.0 ml of total antioxidant reagent, incubated at 95°C for 90 min under dark conditions, cooled to room temperature, and absorbance measured at 695 nm. A reagent blank (total antioxidant reagent plus water) and a sample blank (extract plus water without reagent) were included to eliminate background interference. An ascorbic acid standard curve (0–200 µg ml^-^¹) was used for calibration, and results were expressed as µg ascorbic acid equivalent (AAE) g^-^¹ FW. All assays were carried out in 96-well microtiter plates, with blanks and standards included in each run, and the mean of the blank-corrected absorbance values from three replicates was used for calculations.

#### Characterization of root nodulating bacteria

##### Phenotypic characterization of RNB

###### Morphology and gram staining of RNB strains

Pure cultures of RNB isolates obtained from the nodules of native leguminous trees were characterized for their colony morphology and Gram reaction (Wiegel, 1981). Colony characteristics, including shape (entire, flat, raised) and the presence or nature of extracellular polysaccharide (EPS) such as mucoid, milky, or translucent slime, were carefully observed and documented. All observations were conducted in triplicate for each isolate, with three independent cultures analyzed to ensure reproducibility and accuracy of the data. For Gram staining, a thin smear of actively growing bacterial culture was prepared on a sterile glass slide using standard aseptic techniques. The smear was air-dried, heat-fixed, and initially stained with crystal violet for 2 min, followed by treatment with iodine solution. Decolorization was performed using 95% ethanol for 30 s, and the smear was subsequently rinsed with distilled water and gently blotted. The Gram-stained preparations were examined under a compound microscope at 40× and 100× magnifications to determine the Gram reaction of each isolate (Somasegaran and Hoben, 1994).

###### Salt tolerance assay

To assess the tolerance of root nodule bacterial isolates against salinity, Yeast Extract Mannitol (YEM) agar plates were prepared with graded concentrations of NaCl (1.0, 2.0, and 3.0 g/100mL; w/v) as described by Nohwar et al. (2019). The media without NaCl served as a positive control. Actively growing bacterial cultures were streaked onto the plates, and incubation was carried out at 28 ± 2°C for 4–5 days. Each treatment was maintained in triplicate to ensure statistical validity. The plates were observed at 24 h intervals to record the growth response of isolates at different salt concentrations. The growth pattern on salt-supplemented plates was compared with that of the control set.

###### pH tolerance

The ability of root nodule bacterial isolates to survive across a wide pH range was evaluated by preparing YEM agar plates adjusted to pH values between 4.0 and 11.0. The pH was adjusted using 1 N HCl or 1 N NaOH, following the procedure described by Cappuccino and Sherman (2007). To maintain stable pH, appropriate buffering agents were incorporated into the media: HOMOPIPES (pH 4.5–5.0), MES (pH 5.5–6.0), HEPES (pH 6.8–8.2), and CHES (pH 9.0–10.0). The plates were streaked with actively growing cultures of each isolate and incubated at 28 ± 2°C for 3–4 days. All treatments were performed in triplicate. Post incubation, the plates were examined for visible colony development, and the growth responses were compared across different pH levels.

###### Acidification/alkalinization response on bromothymol blue medium

The acidification and alkalinization potential of RNB isolates was evaluated in Yeast Extract Mannitol (YEM) broth supplemented with bromothymol blue (BTB, 25 mg L^-^¹) as a pH indicator, with the medium adjusted to pH 6.8 ± 0.1 prior to sterilization to yield a neutral green coloration as described by Shihab and Alkurtany (2023). Actively growing log-phase cultures (1% inoculum) were inoculated into 10 mL aliquots of BTB-supplemented broth and incubated at 30 ± 2°C for 5–6 days with continuous shaking at 100 rpm, while uninoculated tubes served as controls. Each treatment was performed in triplicate, and after incubation the tubes were examined for color change, where a shift from green to yellow indicated acid production, a shift to deep blue indicated alkalinization, and retention of green signified neutral activity.

###### Intrinsic antibiotic resistance pattern

The intrinsic antibiotic resistance profiles of RNB isolates were evaluated using the Kirby–Bauer disc diffusion assay as described by Cappuccino and Sherman (2008) with minor modifications. Actively growing broth cultures were spread uniformly on Yeast Extract Mannitol Agar (YEMA) plates, and Hi-Media octa-discs containing antibiotics of defined concentrations were aseptically placed on the surface. The plates were incubated at 28 ± 2°C for 35–40 h, after which the diameters of the inhibition zones surrounding each antibiotic disc were measured in millimeters. The antibiotic panel included Ceftriaxone (CTR, 30 µg), Gentamicin (GEN, 10 µg), Co-trimoxazole (COT, 25 µg), Levofloxacin (LE, 5 µg), Netillin (NET, 30 µg), Tetracycline (TE, 30 µg), Amoxyclav (AMC, 30 µg), Ofloxacin (OF, 5 µg), Amikacin (AK, 10 µg), Carbenicillin (CB, 100 µg), Ciprofloxacin (CIP, 10 µg), Kanamycin (K, 30 µg), Nitrofurantoin (NIT, 300 µg), Streptomycin (S, 10 µg), and Co-trimazine (CM, 30 µg). The resulting inhibition profiles were used to determine the intrinsic antibiotic resistance pattern of the isolates.

##### Biochemical characterization of RNB

###### Nitrate reductase activity

The nitrate-reducing ability of RNB isolates was determined using nitrate broth (Bhusal and Muriana, 2021). Actively growing cultures were inoculated into sterile nitrate broth tubes and incubated at 28 ± 2°C with continuous agitation (100 rpm) for 48 h. After incubation, five drops each of sulfanilic acid and α-naphthylamine were added sequentially. The development of a pink coloration indicated reduction of nitrate (NO_3_^-^) to nitrite (NO_2_^-^). Tubes that remained colorless were further treated with zinc dust to confirm nitrate reduction. Appearance of pink color after zinc addition indicated absence of nitrate reductase activity, while no color change confirmed complete reduction of nitrate to ammonia. Each isolate was tested in triplicate to ensure statistical reliability of the observations.

###### Catalase activity

Catalase production was determined by incubating RNB isolates in Yeast Extract Mannitol (YEM) broth at 28 ± 2°C for 48 h. Following incubation, 4–5 drops of 3% hydrogen peroxide were added to the cultures. Immediate effervescence due to oxygen release was recorded as positive, while absence of bubbling was scored as negative (Isokar et al., 2024). Each assay was carried out in triplicate for accuracy.

###### Oxidase activity

The capacity of isolates to produce cytochrome oxidase was assessed using N, N-dimethyl-p-phenylenediamine impregnated oxidase discs (Ortíz et al., 2020). Pure colonies grown on YEM agar at 30°C for 48 h were tested by applying the discs to the colonies. Rapid development of dark blue coloration within 4–5 s indicated a positive reaction, while no color change was considered negative. Tests were conducted in triplicate to ensure reliability.

###### Amylase production

Amylase activity was assessed on starch agar medium (Fernandes Júnior et al., 2012). Actively growing isolates were spot-inoculated and incubated at 30°C for 48 h. Following incubation, Gram’s iodine solution was applied to the plates. The formation of a distinct halo zone around the colonies against a dark blue background confirmed starch hydrolysis and amylase production, while uniform staining without halo formation was indicative of negative activity. Each isolate was tested in triplicate to ensure statistical significance.

###### Gelatinase activity

Gelatinase production was evaluated in nutrient gelatin broth tubes inoculated with RNB isolates and incubated at 28°C under shaking conditions (120 rpm) for 4–5 days. Post incubation inoculated and uninoculated control tubes were transferred to 4°C for 30 min. Cultures that remained liquefied after refrigeration were considered positive for gelatinase activity, whereas solidification of the medium confirmed the absence of gelatin hydrolysis (Park et al., 2024). Experiments for each isolate were performed in three replicates to ensure reproducibility and statistical validity.

##### Plant growth-promoting characterization of RNB

###### Ammonia production

The potential of RNB isolates to produce ammonia (NH_4_^+^) was evaluated using peptone water. Actively growing bacterial cultures were inoculated into sterile peptone water tubes and incubated at 30 ± 2°C under continuous shaking at 100 rpm for 5 days. These assays were conducted in triplicate for each isolate to confirm consistency and statistical accuracy Following incubation, a few drops of Nessler’s reagent were added to each tube. The appearance of a muddy yellow coloration indicated positive ammonia production, whereas absence of color change was recorded as negative (Cappuccino and Sherman, 2008).

###### Indole-3-acetic acid production

IAA production by RNB isolates was determined colorimetrically following the method of Gordon and Weber (1951). YEM broth supplemented with L-tryptophan (1 mM), in triplicate for each isolate, was inoculated with actively growing cultures and incubated in the dark at 30 ± 2°C with shaking (100 rpm) for 5 days. After incubation, cultures were centrifuged at 10,000 rpm for 10 min, and 2 mL of the supernatant was mixed with 2–3 drops of ortho-phosphoric acid, followed by 4 mL of Salkowski reagent (1 mL of 0.5 M FeCl_3_ in 50 mL H_2_O + 35% perchloric acid). Tubes were vortexed and incubated in darkness for 30 min. Development of a pink color indicated IAA production. Optical density was measured at 530 nm using a spectrophotometer, and values were compared against a reagent blank.

###### Phosphate solubilization

The phosphate-solubilizing ability of RNB strains was evaluated on Pikovskaya’s (PVK) agar. Actively growing cultures were spot-inoculated on PVK plates and incubated at 30 ± 2°C for 5–7 days. Each isolate was assessed in triplicate**, t**he formation of a clear halo surrounding the colonies indicated solubilization of inorganic phosphate via organic acid or phosphatase activity. The diameter of the halo was measured in millimeters to quantify the solubilization potential (Nautiyal, 1999).

###### Cellulase activity

Cellulase production was assessed using carboxymethyl cellulose (CMC) agar plates containing Congo red dye. Pure RNB cultures were spot-inoculated and incubated at 30 ± 2°C for 5–10 days. Formation of a clear zone around colonies after staining indicated enzymatic degradation of cellulose, confirming cellulase activity (Kasana et al., 2008).

###### Chitinase activity

Chitinase activity was determined using colloidal chitin agar plates. Activated RNB cultures were spot-inoculated in triplicate manner onto the medium and incubated at 30 ± 2°C for 5–10 days. After incubation, plates were flooded with Gram’s iodine solution, and the formation of clear zones around colonies indicated chitin hydrolysis and positive chitinase activity (Siddiqui et al., 2021).

###### Pectinase activity

The production of pectinase by RNB isolates was evaluated on pectin agar plates. Actively growing cultures were spot-inoculated and incubated at 30 ± 2°C for 5–7 days. After incubation, Gram’s iodine solution was applied to the plates. Clear halo zones around the colonies indicated enzymatic degradation of pectin, confirming pectinase production, while unaltered deep blue coloration denoted negative results (Ronald and James, 2006).

### Statistical analysis

All data collected from the pot experiment, nodule characterization, and root-nodulating bacterial (RNB) isolate assessments were subjected to comprehensive statistical evaluation to ensure accuracy, consistency, and reliability of the results. Descriptive statistics, including mean, standard deviation (SD), and standard error (SE), were computed for all measured parameters covering soil physico-chemical properties (pH, electrical conductivity, organic carbon, available N, P, K), seedling growth traits (plant height, number of leaves, stem diameter, and root length), nodule morphology (size, shape, pigmentation), and biochemical constituents (phenol, tannin, FRAP, and total antioxidant content). Prior to inferential analyses, the Shapiro–Wilk test was employed to examine data normality. The results confirmed that most variables followed a normal distribution (p > 0.05), validating the use of parametric tests. The assumption of homogeneity of variances across treatment groups was further verified using Levene’s test for homoscedasticity, which indicated equal variance (p > 0.05) among groups, thereby justifying the application of one-way analysis of variance (ANOVA). Subsequently, ANOVA was performed to detect significant differences (p < 0.05) among treatments or isolates for all evaluated parameters. When significant effects were observed, mean separation was conducted using the Least Significant Difference (LSD) test at the 5% probability level to determine pairwise differences among treatment means. To understand the degree and direction of relationships among variables, Pearson’s correlation coefficient (r) was calculated between soil nutrient parameters, nodule biochemical traits, and seedling growth attributes of *P. pinnata*. This helped to identify significant associations (positive or negative) between soil fertility indices, nodule metabolism, and plant performance. Furthermore, multiple linear regression analysis was carried out to quantify the relative contribution of individual soil and biochemical parameters to seedling growth responses (plant height, number of leaves, stem diameter, and root length). Regression determination coefficients (R²) were used to evaluate the strength and predictive power of the models, elucidating how variations in nutrient status and nodule biochemistry collectively influence growth performance. To refine and identify the most influential predictors, stepwise regression analysis was also applied, which iteratively included or excluded variables based on their statistical significance, producing optimized models for each growth parameter. In addition, Principal Component Analysis (PCA) was employed to explore multivariate relationships among soil, biochemical, and growth variables, and to visualize clustering patterns of RNB isolates based on their combined traits. The first two principal components (Dim1 and Dim2) explained the majority of total variance, effectively separating isolates according to their biochemical and growth performance profiles. Complementarily, hierarchical cluster analysis (HCA) using Ward’s linkage and Euclidean distance metrics was performed to classify the twenty sampling sites into distinct groups based on their soil fertility gradients and biochemical characteristics. All statistical analyses were performed using R statistical software (version 4.5.1). Data were presented as mean ± standard error (SE) of three replicates. This rigorous statistical approach ensured reliable interpretation of the data and provided an integrated understanding of the associations (correlation) and contributions (regression) between soil nutrient status, biochemical composition of root nodules, and plant growth responses of *P. pinnata*.

## Results

### Soil nutritional status

**Figure 1:**
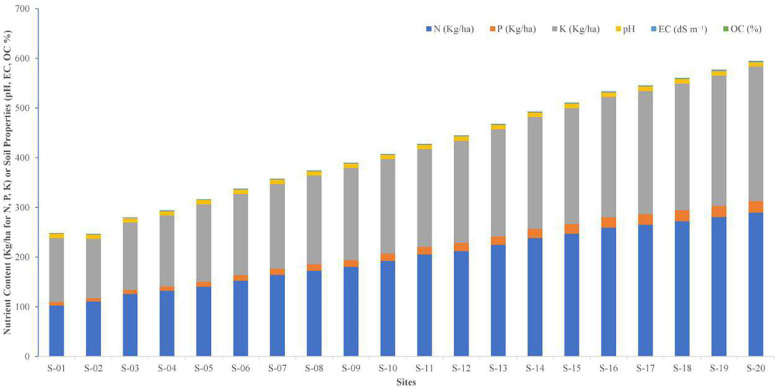
Variation in soil physico-chemical properties (pH, EC, SOC) and available nutrient content (N, P, K) across 20 rhizospheric sites of *P. pinnata* in the arid regions of western Rajasthan.

Soil nutrient content and physico-chemical properties varied significantly across the 20 locations. Nitrogen content ranged from 102.3 ± 3.4 kg/ha at S-01 to 289.5 ± 3.8 kg/ha at S-20, with sites S-16 to S-20 exhibiting the highest values (F = 58.69, p < 0.001; LSD = 1.25). Available phosphorus varied between 6.9 ± 0.3 kg/ha (S-02) and 23.2 ± 0.5 kg/ha (S-20), with a significant increase observed in sites S-16 to S-20 (F = 10.25, p < 0.001; LSD = 0.08). Potassium content was lowest at S-02 (119.6 ± 3.8 kg/ha) and highest at S-20 (270.6 ± 4.1 kg/ha), showing a significant progressive increase across sites (F = 114.65, p < 0.001; LSD = 2.24). Soil pH ranged from 8.2 ± 0.1 (S-03) to 9.1 ± 0.1 (S-19 and S-20), with sites S-11 to S-20 recording significantly higher pH values (F = 41.23, p < 0.001; LSD = 0.04). Although these differences are statistically significant, the narrow pH range (8.2–9.1) is ecologically marginal, as *P. pinnata* is known to tolerate slightly alkaline soils. Therefore, these minor variations are unlikely to have a substantial impact on seedling growth or nodule formation, suggesting that other soil factors, such as nutrient availability or organic matter content, may play a more decisive role in influencing plant performance across sites. Electrical conductivity (EC) increased from 1.18 ± 0.03 dS m^-^¹ at S-02 to 1.89 ± 0.04 dS m^-^¹ at S-20, with sites S-14 to S-20 showing significantly higher EC levels (F = 25.65, p < 0.001; LSD = 0.001). Although statistically significant, these EC values fall within the range considered non-saline according to FAO and ICAR soil standards (<2 dS m^-^¹), indicating that salinity is unlikely to adversely affect *P. pinnata* growth. Soil organic carbon (SOC) content also showed a significant increasing trend, ranging from 0.21 ± 0.01% (S-02) to 0.53 ± 0.02% (S-20) (F = 31.33, p < 0.001; LSD = 0.001). The higher SOC at sites S-16 to S-20 suggests better soil fertility and improved water- and nutrient-holding capacity, which can enhance seedling establishment and rhizobial activity. Sites S-16 to S-20 consistently exhibited higher nutrient contents and soil fertility indicators compared to the other locations, highlighting spatial variability in the arid region soils of western Rajasthan (**Supplementary Table 1**, **Figure 1**).

### Growth performance of *P. pinnata* seedlings

**Figure 2:**
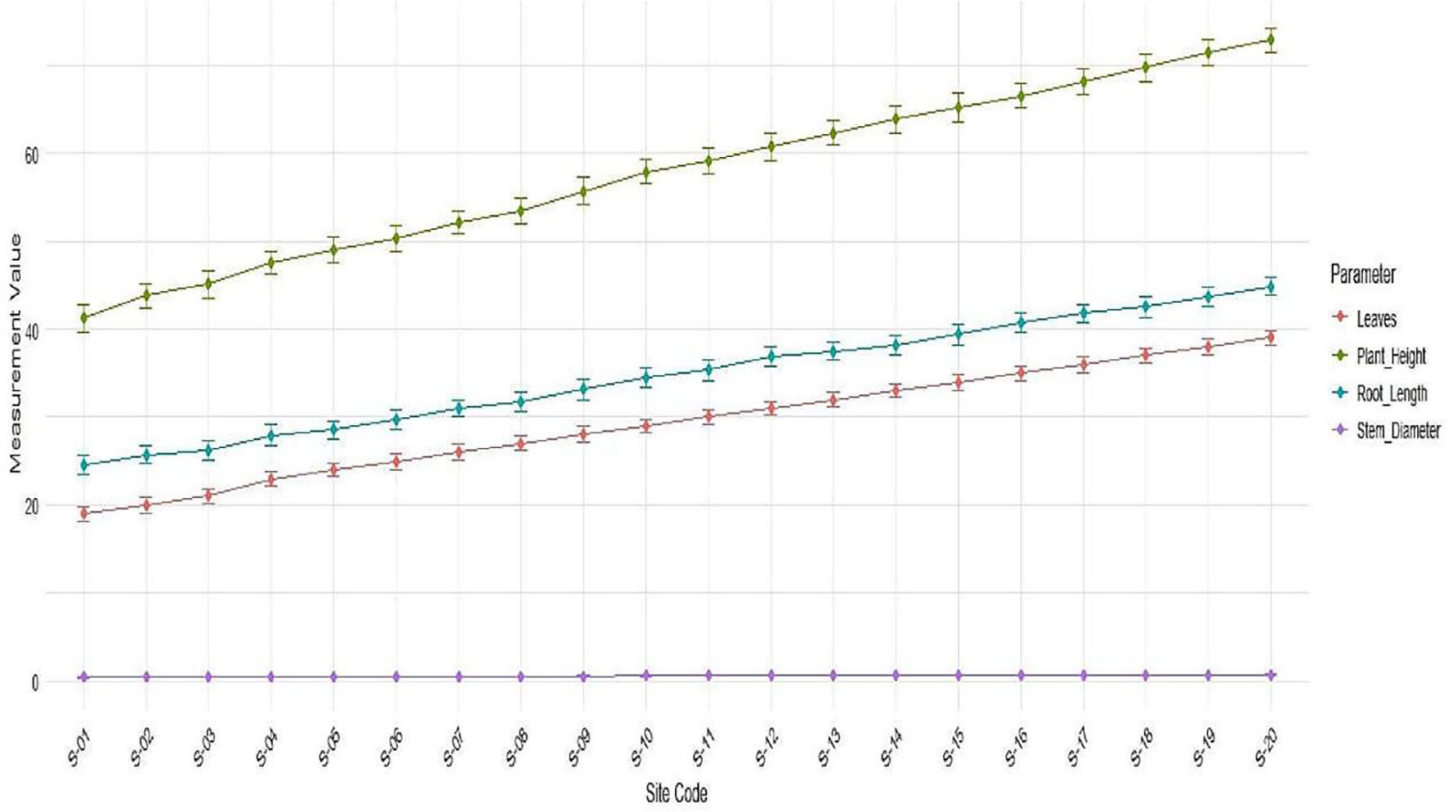
Variation in growth performance (plant height, number of leaves, stem diameter, and root length) of *P. pinnata* seedlings across 20 rhizospheric sites in the arid regions of western Rajasthan.

At 90 days, plant height of *P. pinnata* seedlings showed a significant increase across sites, with S-01 (41.2 ± 1.5 cm) and S-02 (43.8 ± 1.4 cm) being the lowest, whereas seedlings from S-19 (71.4 ± 1.5 cm) and S-20 (72.8 ± 1.4 cm) exhibited the highest growth (F = 120.36, p < 0.001; LSD = 0.98). Similarly, the number of leaves was significantly higher in seedlings from sites S-18 to S-20 (37.0 ± 0.8 to 39.0 ± 0.8) compared to S-01 to S-03 (19.0 ± 0.8 to 21.0 ± 0.8) (F = 95.63, p < 0.001; LSD = 0.43). Stem diameter also increased significantly with site variation, where S-01 (0.42 ± 0.02 mm) and S-02 (0.44 ± 0.02 mm) had the lowest values, whereas seedlings from S-19 (0.76 ± 0.02 cm) and S-20 (0.78 ± 0.02 cm) recorded the highest (F = 42.7, p < 0.001; LSD = 0.001). Furthermore, root length followed a similar trend, showing a significant increase from S-01 (24.5 ± 1.1 cm) to S-20 (44.8 ± 1.0 cm) (F = 131.57, p < 0.001; LSD = 0.15). Moreover, seedlings from sites S-16 to S-20 consistently exhibited superior growth in all measured parameters compared to other sites, indicating a strong influence of rhizospheric soil conditions on seedling performance. However, seedlings from S-01 to S-05 showed comparatively lower growth, reflecting possible limitations in soil fertility and nutrient availability (**Supplementary Table 2**, **Figure 2**).

### Morphological nodule characterization

The nodule size varied among the isolates, with lengths ranging from 2.5 mm (PP-07) to 4.2 mm (PP-09) and diameters from 1.6 mm (PP-20) to 2.7 mm (PP-09). The nodules exhibited diverse shapes, including spherical, elongated, and irregular forms, while their external coloration ranged from light brown, brown, and dark brown to yellowish brown and brownish green. Internally, all nodules displayed a pink to pinkish-red coloration, indicating active nitrogen fixation. Notably, all isolates produced effective nodules, suggesting a consistent symbiotic potential across the isolates for promoting nitrogen fixation in *P. pinnata* seedlings. Spherical nodules were predominant among the isolates, particularly in PP-01, PP-03, PP-06, PP-09, PP-11, PP-14, PP-16, and PP-19, while elongated nodules were observed in PP-02, PP-05, PP-08, PP-12, PP-15, and PP-18. Irregular nodules were recorded in PP-04, PP-07, PP-10, PP-13, PP-17, and PP-20. These findings highlight the morphological diversity of nodules among the *Rhizobium* isolates while confirming their effectiveness in symbiotic nitrogen fixation under arid conditions (**Table 2**).

**Table 2:** Morphological characteristics and effectiveness of root nodules formed by Rhizobia isolates (PP-01 to PP-20) associated with *Pongamia pinnata* in arid regions of western Rajasthan.

Isolate code	Size (mm) (Length × Diameter)	Shape	External colour	Internal colour	Nodule effectiveness
PP-01	3.2 × 2.1	Spherical	Light Brown	Pinkish Red	Effective
PP-02	2.8 × 1.9	Elongated	Brown	Pink	Effective
PP-03	4.0 × 2.5	Spherical	Dark Brown	Pink	Effective
PP-04	3.5 × 2.0	Irregular	Yellowish Brown	Pinkish Red	Effective
PP-05	2.9 × 1.8	Elongated	Brown	Pink	Effective
PP-06	3.8 × 2.3	Spherical	Light Brown	Pinkish Red	Effective
PP-07	2.5 × 1.6	Irregular	Brownish Green	Pink	Effective
PP-08	3.6 × 2.2	Elongated	Brown	Pinkish Red	Effective
PP-09	4.2 × 2.7	Spherical	Dark Brown	Pink	Effective
PP-10	2.7 × 1.7	Irregular	Yellowish Brown	Pink	Effective
PP-11	3.3 × 2.0	Spherical	Light Brown	Pinkish Red	Effective
PP-12	3.0 × 1.9	Elongated	Brown	Pink	Effective
PP-13	2.6 × 1.8	Irregular	Brownish Yellow	Pinkish Red	Effective
PP-14	3.9 × 2.4	Spherical	Dark Brown	Pink	Effective
PP-15	2.8 × 1.7	Elongated	Brown	Pinkish Red	Effective
PP-16	3.7 × 2.2	Spherical	Light Brown	Pink	Effective
PP-17	2.9 × 1.8	Irregular	Yellowish Brown	Pinkish Red	Effective
PP-18	4.1 × 2.6	Elongated	Brown	Pink	Effective
PP-19	3.4 × 2.1	Spherical	Dark Brown	Pinkish Red	Effective
PP-20	2.7 × 1.6	Irregular	Light Brown	Pink	Effective

### Biochemical nodule characterization

**Figure 3:**
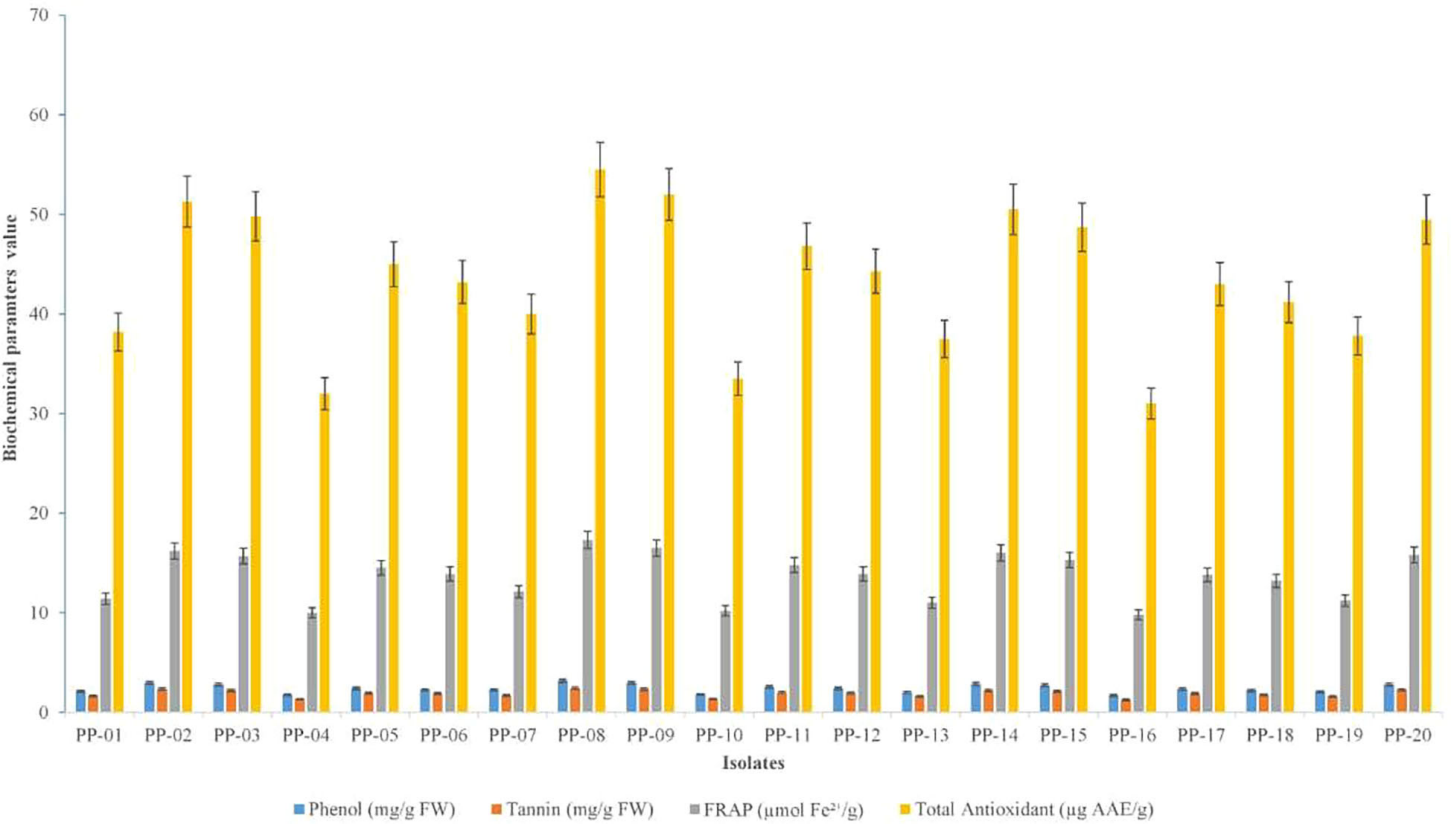
Variation in phenol, tannin, FRAP, and total antioxidant content in root nodules of *P. pinnata* isolates (PP-01 to PP-20) collected from arid region sites of western Rajasthan.

The biochemical characterization of root nodules of *P. pinnata* isolates (PP-01 to PP-20) revealed significant variation in phenol, tannin, FRAP, and total antioxidant content among the isolates. Phenol content ranged from 1.70 ± 0.08 mg/g FW in PP-16 to 3.15 ± 0.09 mg/g FW in PP-08, with isolates PP-02, PP-03, PP-05, PP-06, PP-08, PP-09, PP-11, PP-12, PP-14, PP-15, PP-17, PP-18, and PP-20 showing significantly higher levels compared to PP-01, PP-04, PP-07, PP-10, PP-13, PP-16, and PP-19 (F = 45.65, p < 0.001; LSD = 0.09). Similarly, tannin content was highest in PP-02 (2.34 ± 0.08 mg/g FW) and PP-08 (2.42 ± 0.08 mg/g FW), whereas PP-16 (1.25 ± 0.06 mg/g FW) and PP-04 (1.30 ± 0.06 mg/g FW) recorded the lowest values (F = 98.54, p < 0.001; LSD = 0.04). The FRAP values, representing reducing power, varied from 9.8 ± 0.5 µmol Fe²^+^/g in PP-16 to 17.3 ± 0.6 µmol Fe²^+^/g in PP-08 (F = 41.25, p < 0.001; LSD = 0.1), indicating higher antioxidant potential in isolates such as PP-02, PP-03, PP-05, PP-08, PP-09, PP-11, PP-12, PP-14, PP-15, PP-17, PP-18, and PP-20. Total antioxidant content also showed a similar trend, ranging from 31.0 ± 1.1 µg AAE/g in PP-16 to 54.5 ± 1.2 µg AAE/g in PP-08, with isolates PP-02, PP-03, PP-05, PP-08, PP-09, PP-11, PP-12, PP-14, PP-15, PP-17, PP-18, and PP-20 exhibiting significantly higher levels than PP-01, PP-04, PP-07, PP-10, PP-13, PP-16, and PP-19 (F = 53.67, p < 0.001; LSD = 0.87). Isolates PP-02, PP-03, PP-05, PP-08, PP-09, PP-11, PP-12, PP-14, PP-15, PP-17, PP-18, and PP-20 consistently showed superior biochemical attributes, suggesting their strong potential for enhancing the antioxidant and phenolic profile in *P. pinnata* nodules (**Supplementary Table 3**, **Figure 3**).

### Phenotypic characterization of RNB isolates

**Figure 4:**
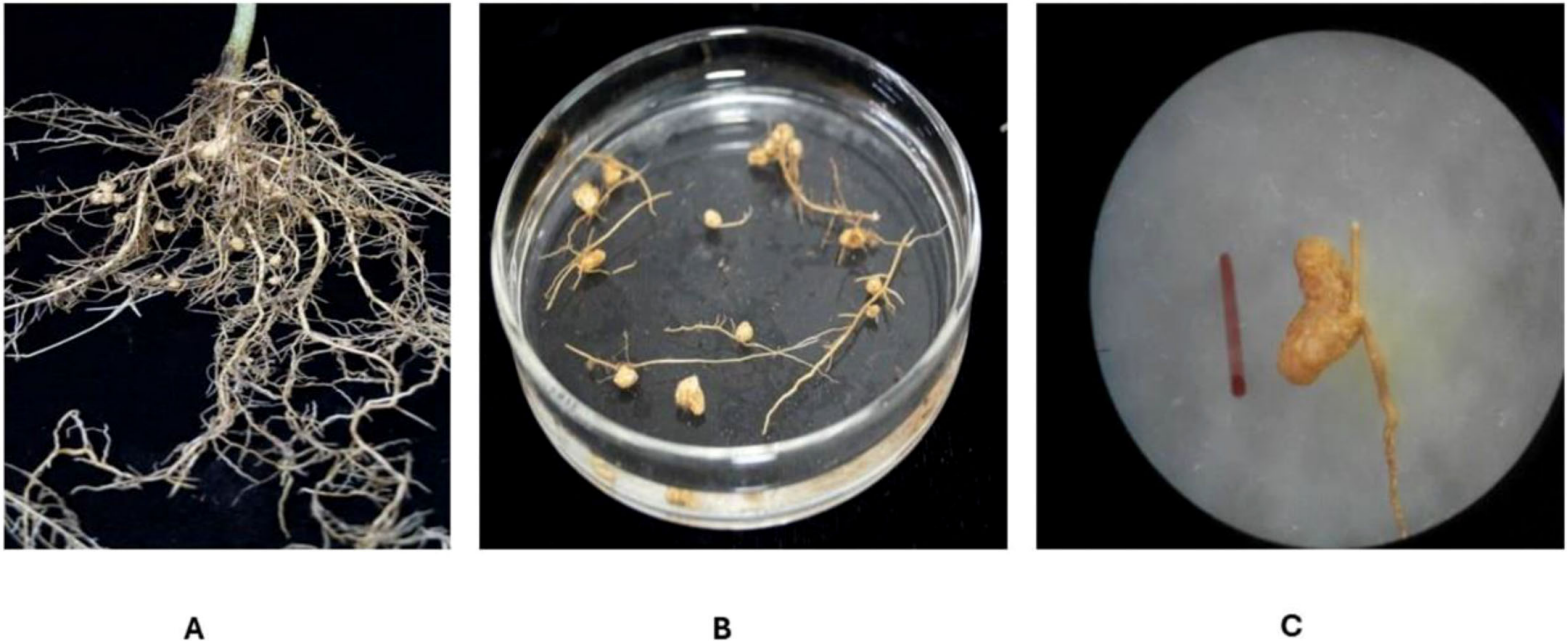
Root nodules on roots. **(A)** Nodules attached along fine lateral roots, illustrating typical distribution and density **(B)** Detached nodules highlighting variations in in size, shape, and surface morphology. **(C)** Close-up of indeterminate, highly branched nodules, showing characteristic elongated architecture typical of perennial legume symbioses.

**Figure 5:**
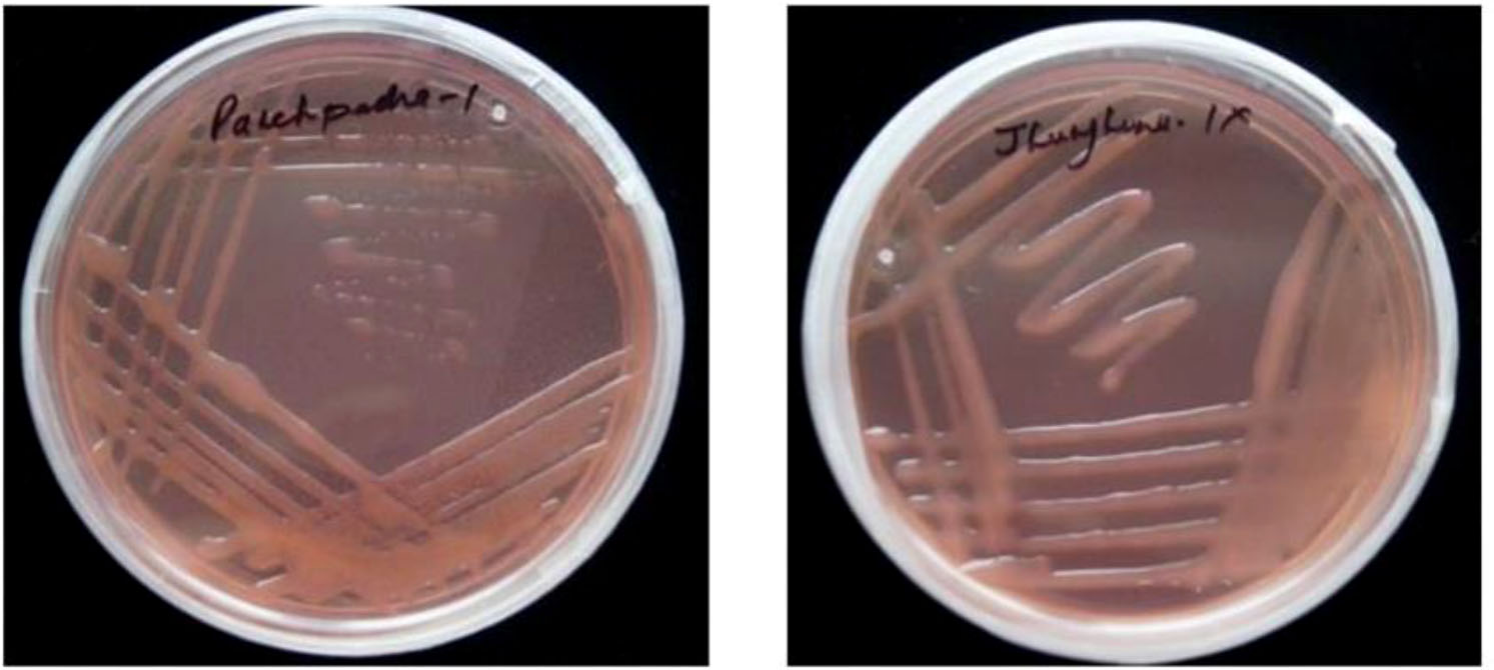
Colonies of Rhizobium isolates on CR-YEMA media after incubation under standard conditions.

All twenty *Rhizobium* isolates (PP-01 to PP-20) obtained from the rhizospheric soil of *P. pinnata* exhibited distinct morphological and physiological characteristics (**Figure 4**, **5**) (**Table 3**). Colonies of all isolates were circular, white in color, slimy in texture, and opaque, with Gram staining confirming that all were Gram-negative. Colony size varied noticeably among the isolates, ranging from 1.0 mm (PP-06, PP-08, PP-13, and PP-14) to 3.0 mm (PP-10 and PP-20), indicating diversity in their growth potential on culture media. The isolates also displayed considerable variation in their salt tolerance. Most isolates were tolerant up to 1% NaCl (172 mM), while PP-02, PP-03, PP-07, PP-14, PP-16, and PP-19 could tolerate up to 2% NaCl (342 mM). The highest salt tolerance of 3% NaCl (513 mM) was observed in isolates PP-05, PP-10, PP-11, PP-13, and PP-18, demonstrating their ability to survive under saline conditions typical of arid soils. All isolates showed a broad pH tolerance, growing well within a range of 5–11, except for a few (PP-01, PP-03, PP-11, PP-13, and PP-15) which were limited to pH 5–10, and PP-09 and PP-17, which grew between pH 6–11. This indicates that the isolates possess substantial adaptability to both acidic and alkaline soil environments. The bromothymol blue (BTB) reaction further revealed variations among the isolates. Acidic reactions were observed in isolates such as PP-01, PP-07, PP-08, PP-09, PP-12, PP-13, PP-15, and PP-19, while PP-02, PP-03, PP-05, PP-10, PP-16, and PP-18 produced an alkaline reaction. Neutral reactions were recorded in PP-04, PP-06, PP-11, PP-14, and PP-17. These findings highlight significant diversity among the *Rhizobium* isolates in terms of colony morphology, salt tolerance, pH adaptability, and BTB reaction, suggesting their potential resilience and functional versatility under the challenging soil conditions of arid regions.

**Table 3:** Salt tolerance, pH range, and bromothymol blue (BTB) reaction of twenty RNB isolates (PP-01 to PP-20) from *Pongamia pinnata*.

Isolate	Colony size(mm)	Colony shape	Colony colour	Gram staining	Texture	Opacity	Gram staining	NaCl (%)	pH range	BTB reaction
PP-01	1.5	Circular	White	Negative	Slimy	Opaque	Gram negative	1% (172 mM)	5–10	Acidic
PP-02	2.5	Circular	White	Negative	Slimy	Opaque	Gram negative	2% (342 mM)	5–11	Alkaline
PP-03	1.5	Circular	White	Negative	Slimy	Opaque	Gram negative	2% (342 mM)	5–10	Alkaline
PP-04	1.5	Circular	White	Negative	Slimy	Opaque	Gram negative	1% (172 mM)	5–11	Neutral
PP-05	1.5	Circular	White	Negative	Slimy	Opaque	Gram negative	3% (513 mM)	5–11	Alkaline
PP-06	1	Circular	White	Negative	Slimy	Opaque	Gram negative	1% (172 mM)	5–11	Neutral
PP-07	2.5	Circular	White	Negative	Slimy	Opaque	Gram negative	2% (342 mM)	5–11	Acidic
PP-08	1	Circular	White	Negative	Slimy	Opaque	Gram negative	1% (172 mM)	5–11	Acidic
PP-09	2.5	Circular	White	Negative	Slimy	Opaque	Gram negative	1% (172 mM)	6–11	Acidic
PP-10	3	Circular	White	Negative	Slimy	Opaque	Gram negative	3% (513 mM)	5–11	Alkaline
PP-11	1.5	Circular	White	Negative	Slimy	Opaque	Gram negative	3% (513 mM)	5–10	Neutral
PP-12	1.5	Circular	White	Negative	Slimy	Opaque	Gram negative	1% (172 mM)	5–11	Acidic
PP-13	1	Circular	White	Negative	Slimy	Opaque	Gram negative	3% (513 mM)	5–10	Acidic
PP-14	1	Circular	White	Negative	Slimy	Opaque	Gram negative	2% (342 mM)	5–11	Neutral
PP-15	2	Circular	White	Negative	Slimy	Opaque	Gram negative	1% (172 mM)	5–10	Acidic
PP-16	1.5	Circular	White	Negative	Slimy	Opaque	Gram negative	2% (342 mM)	5–11	Alkaline
PP-17	2.5	Circular	White	Negative	Slimy	Opaque	Gram negative	1% (172 mM)	6–11	Neutral
PP-18	2	Circular	White	Negative	Slimy	Opaque	Gram negative	3% (513 mM)	5–11	Alkaline
PP-19	2.5	Circular	White	Negative	Slimy	Opaque	Gram negative	2% (342 mM)	5–10	Acidic
PP-20	3	Circular	White	Negative	Slimy	Opaque	Gram negative	1% (172 mM)	5–11	Acidic

### Intrinsic anti-biotic resistance assay of RNB isolates

**Figure 6:**
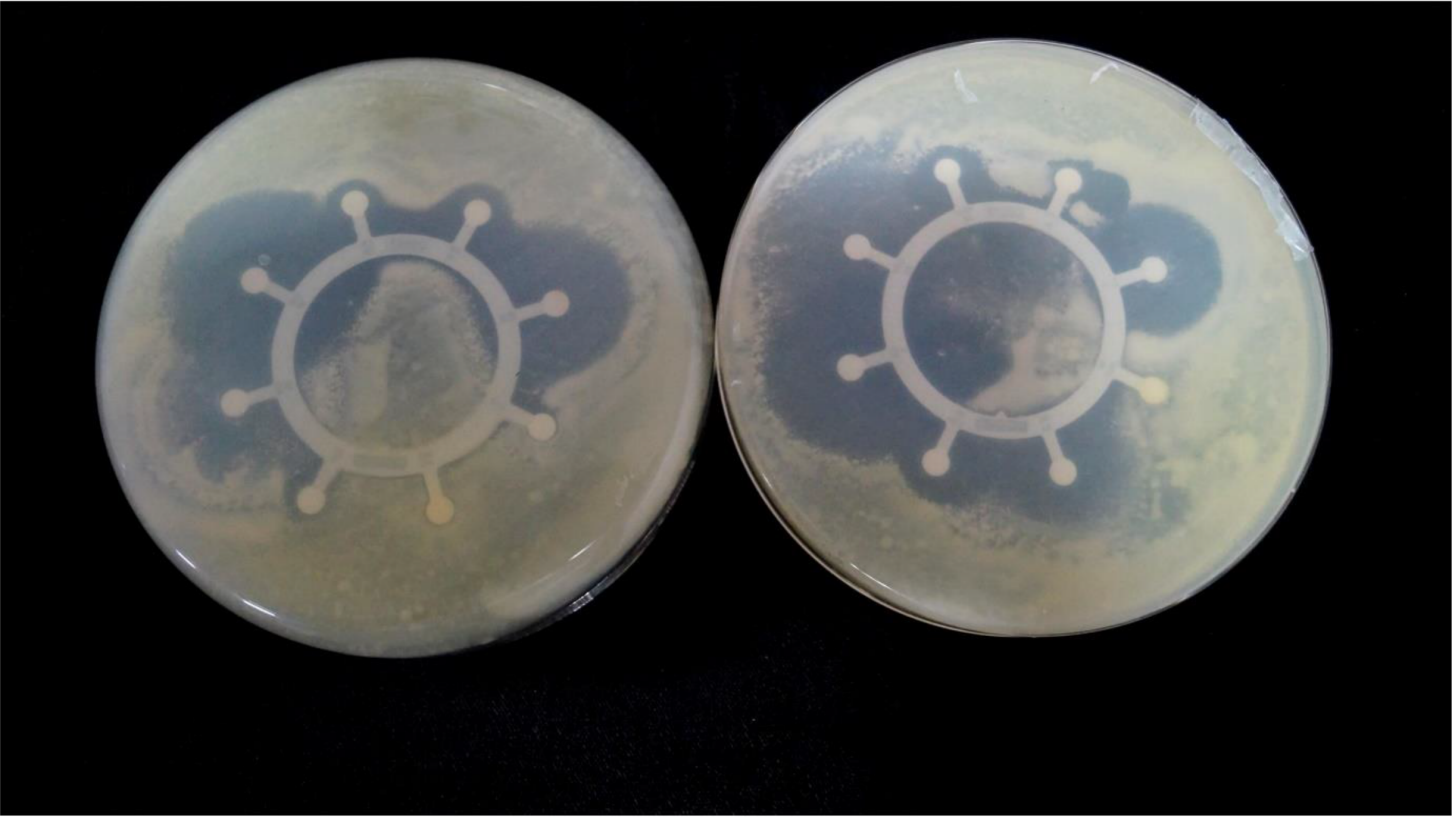
Petri plates showing the growth of *Rhizobium* isolates in the presence of selected antibiotics, demonstrating their intrinsic resistance and sensitivity patterns under standardized conditions.

The intrinsic antibiotic resistance of twenty *Rhizobium* isolates (PP-01 to PP-20) associated with *P. pinnata* exhibited significant variation across the tested antibiotics. The zone of inhibition for chloramphenicol (CTR) ranged from 10 ± 0.5 mm in PP-02, PP-05, PP-10, and PP-15 to 19 ± 0.7 mm in PP-04, PP-07, and PP-13 (F = 68.59, p < 0.001; LSD = 0.12). Gentamicin (GEN) showed the highest inhibition in PP-11 (40 ± 0.8 mm), while the lowest was recorded in PP-03 (14 ± 0.5 mm) (F = 110.25, p < 0.001; LSD = 0.24). Cotrimoxazole (COT) resistance varied between 11 ± 0.5 mm in PP-13 and 40 ± 0.8 mm in PP-02, PP-08, PP-11, and PP-12 (F = 45.68, p < 0.001; LSD = 0.31). Levofloxacin (LE) exhibited consistently high inhibition (35–40 mm) across all isolates (F = 98.69, p < 0.001; LSD = 0.16). Netilmicin (NET) resistance ranged from 10 ± 0.5 mm in PP-11 to 30 ± 0.7 mm in PP-05 (F = 44.36, p < 0.001; LSD = 0.10), while tetracycline (TE) zones varied between 19 ± 0.5 mm in PP-12 to 40 ± 0.7 mm in PP-17 (F = 25.86, p < 0.001; LSD = 0.37). Amoxicillin-clavulanic acid (AMC) showed inhibition from 10 ± 0.5 mm in PP-05 to 18 ± 0.5 mm in PP-20 (F = 102.74, p < 0.001; LSD = 0.22). Similarly, ofloxacin (OF) zones ranged from 20 ± 0.5 mm in PP-12 to 40 ± 0.6 mm in PP-09 and PP-17 (F = 36.56, p < 0.001; LSD = 0.36), and amikacin (AK) inhibition ranged from 16 ± 0.5 mm in PP-03 to 40 ± 0.6 mm in PP-11 (F = 94.28, p < 0.001; LSD = 0.27). Cefotaxime (CB) resistance varied from 10 ± 0.5 mm in PP-10 and PP-12 to 40 ± 0.7 mm in PP-01, PP-03, PP-05, and PP-08 (F = 52.63, p < 0.001; LSD = 0.19). Ciprofloxacin (CIP) inhibition ranged from 11 ± 0.5 mm in PP-02 and PP-03 to 40 ± 0.7 mm in multiple isolates including PP-01, PP-05, and PP-08 (F = 111.33, p < 0.001; LSD = 0.34). Chloramphenicol (CM) zones varied between 10 ± 0.5 mm in PP-06 and 40 ± 0.6 mm in PP-04 and PP-08 (F = 129.63, p < 0.001; LSD = 0.44). Kanamycin (K) resistance ranged from 11 ± 0.5 mm in PP-03 to 24 ± 0.6 mm in PP-10 and PP-13 (F = 75.96, p < 0.001; LSD = 0.38), while nitrofurantoin (NIT) inhibition ranged from 10 ± 0.5 mm in PP-08 and PP-12 to 40 ± 0.7 mm in PP-02, PP-05, and PP-13 (F = 90.25, p < 0.001; LSD = 0.27). Streptomycin (S) zones ranged from 10 ± 0.5 mm in PP-03 to 40 ± 0.7 mm in PP-10, PP-11, and PP-15 (F = 64.71, p < 0.001; LSD = 0.14). Isolates PP-04, PP-07, PP-08, PP-09, PP-11, PP-13, and PP-17 consistently exhibited higher resistance against most tested antibiotics, whereas PP-02, PP-03, PP-05, PP-10, and PP-15 displayed comparatively lower resistance, indicating intrinsic variability in antibiotic tolerance among the *Rhizobium* isolates (**Supplementary Table 4**, **Figure 6**).

### Biochemical characterization of RNB isolates

The biochemical characterization of twenty RNB isolates (PP-01 to PP-20) associated with *P. pinnata* revealed distinct enzymatic and metabolic profiles. Nitrate reduction was positive in the majority of isolates, including PP-01, PP-02, PP-04, PP-05, PP-07, PP-08, PP-10, PP-11, PP-13, PP-14, PP-16, PP-17, PP-19, and PP-20, whereas PP-03, PP-06, PP-09, PP-12, PP-15, and PP-18 were negative. Catalase activity was observed in seven isolates (PP-02, PP-05, PP-08, PP-11, PP-14, and PP-17), while the remaining isolates were catalase-negative. All isolates exhibited positive oxidase activity, indicating a consistent presence of cytochrome c oxidase across the strains. Gelatin hydrolysis was absent in all twenty isolates, suggesting a lack of extracellular protease activity under the tested conditions. Amylase activity was detected only in PP-04, PP-09, and PP-19, while the other isolates showed negative results, reflecting limited starch-degrading capability. The absence of gelatinase indicates that these Rhizobium isolates may have restricted ability to degrade complex proteins in the soil, which could limit nutrient mobilization from organic matter but may also reduce metabolic cost and help maintain symbiotic efficiency. Similarly, low amylase activity suggests that starch utilization in the rhizosphere is limited to a few isolates, potentially constraining rapid growth in nutrient-rich microenvironments but allowing specialization in root-associated niches. Collectively, these biochemical traits may influence the survival and competitiveness of the isolates in the rhizosphere, with isolates possessing amylase activity potentially better adapted to exploit transient carbon sources while maintaining stable symbiotic interactions with *P. pinnata*. These findings demonstrate variability in biochemical traits among the Rhizobium isolates, which may influence their metabolic adaptability and symbiotic efficiency with *P. pinnata* (**Table 4**).

**Table 4:** Biochemical characterization of 20 RNB isolates for nitrate reduction, catalase activity, oxidase activity, gelatin hydrolysis, and amylase activity.

Isolates	Nitrate reduction	Catalase activity	Oxidase activity	Gelatin hydrolysis	Amylase activity
PP-01	Positive	Negative	Positive	Negative	Negative
PP-02	Positive	Positive	Positive	Negative	Negative
PP-03	Negative	Negative	Positive	Negative	Negative
PP-04	Positive	Negative	Positive	Negative	Positive
PP-05	Positive	Positive	Positive	Negative	Negative
PP-06	Negative	Negative	Positive	Negative	Negative
PP-07	Positive	Negative	Positive	Negative	Negative
PP-08	Positive	Positive	Positive	Negative	Negative
PP-09	Negative	Negative	Positive	Negative	Positive
PP-10	Positive	Negative	Positive	Negative	Negative
PP-11	Positive	Positive	Positive	Negative	Negative
PP-12	Negative	Negative	Positive	Negative	Negative
PP-13	Positive	Negative	Positive	Negative	Negative
PP-14	Positive	Positive	Positive	Negative	Negative
PP-15	Negative	Negative	Positive	Negative	Negative
PP-16	Positive	Negative	Positive	Negative	Negative
PP-17	Positive	Positive	Positive	Negative	Negative
PP-18	Negative	Negative	Positive	Negative	Negative
PP-19	Positive	Negative	Positive	Negative	Positive
PP-20	Positive	Negative	Positive	Negative	Negative

### Plant growth-promoting activities

The plant growth-promoting biochemical and enzymatic activities of twenty RNB isolates (PP-01 to PP-20) associated with *P. pinnata* showed notable variability. Phosphate solubilization was observed only in PP-04, while all other isolates were negative, indicating limited phosphorus-mobilizing capability among the strains. Indole-3-acetic acid (IAA) production was consistently positive across all isolates, suggesting a strong potential to enhance plant growth through auxin-mediated pathways. Ammonia production was detected in only three isolates: PP-05, PP-12, and PP-05, indicating that most strains do not contribute significantly to nitrogen enrichment via ammonia secretion. Cellulase activity was absent in all isolates, showing no capacity for cellulose degradation under the tested conditions. Chitinase activity was observed only in PP-03 and PP-08, suggesting limited antifungal potential. Pectinase activity was positive in only PP-05, indicating restricted ability for pectin degradation. These observations highlight a limitation of the isolates in terms of biocontrol potential, as the low occurrence of chitinase and pectinase activities suggests that most strains may not effectively suppress fungal pathogens in the rhizosphere. Overall, IAA production is a common trait among the Rhizobium isolates, other plant growth-promoting traits such as phosphate solubilization, ammonia production, and enzymatic activities are strain-specific, reflecting their differential potential to support plant growth and soil nutrient cycling (**Table 5**).

**Table 5:** Biochemical and enzymatic activities of 20 RNB isolates, including phosphate solubilization, IAA production, ammonia production, cellulase, chitinase, and pectinase activities.

Isolates	Phosphate solubilization	IAA production	Ammonia production	Cellulase activity	Chitinase activity	Pectinase activity
PP-01	Negative	Positive	Negative	Negative	Negative	Negative
PP-02	Negative	Positive	Negative	Negative	Negative	Negative
PP-03	Negative	Positive	Negative	Negative	Positive	Negative
PP-04	Positive	Positive	Negative	Negative	Negative	Negative
PP-05	Negative	Positive	Positive	Negative	Negative	Positive
PP-06	Negative	Positive	Negative	Negative	Negative	Negative
PP-07	Negative	Positive	Negative	Negative	Negative	Negative
PP-08	Negative	Positive	Negative	Negative	Positive	Negative
PP-09	Negative	Positive	Negative	Negative	Negative	Negative
PP-10	Negative	Positive	Negative	Negative	Negative	Negative
PP-11	Negative	Positive	Negative	Negative	Negative	Negative
PP-12	Negative	Positive	Positive	Negative	Negative	Negative
PP-13	Negative	Positive	Negative	Negative	Negative	Negative
PP-14	Negative	Positive	Negative	Negative	Negative	Negative
PP-15	Negative	Positive	Negative	Negative	Negative	Negative
PP-16	Negative	Positive	Negative	Negative	Negative	Negative
PP-17	Negative	Positive	Negative	Negative	Negative	Negative
PP-18	Negative	Positive	Negative	Negative	Negative	Negative
PP-19	Negative	Positive	Negative	Negative	Negative	Negative
PP-20	Negative	Positive	Negative	Negative	Negative	Negative

### Association and contribution between soil nutrient status, plant growth response and biochemical composition root nodules

**Figure 7:**
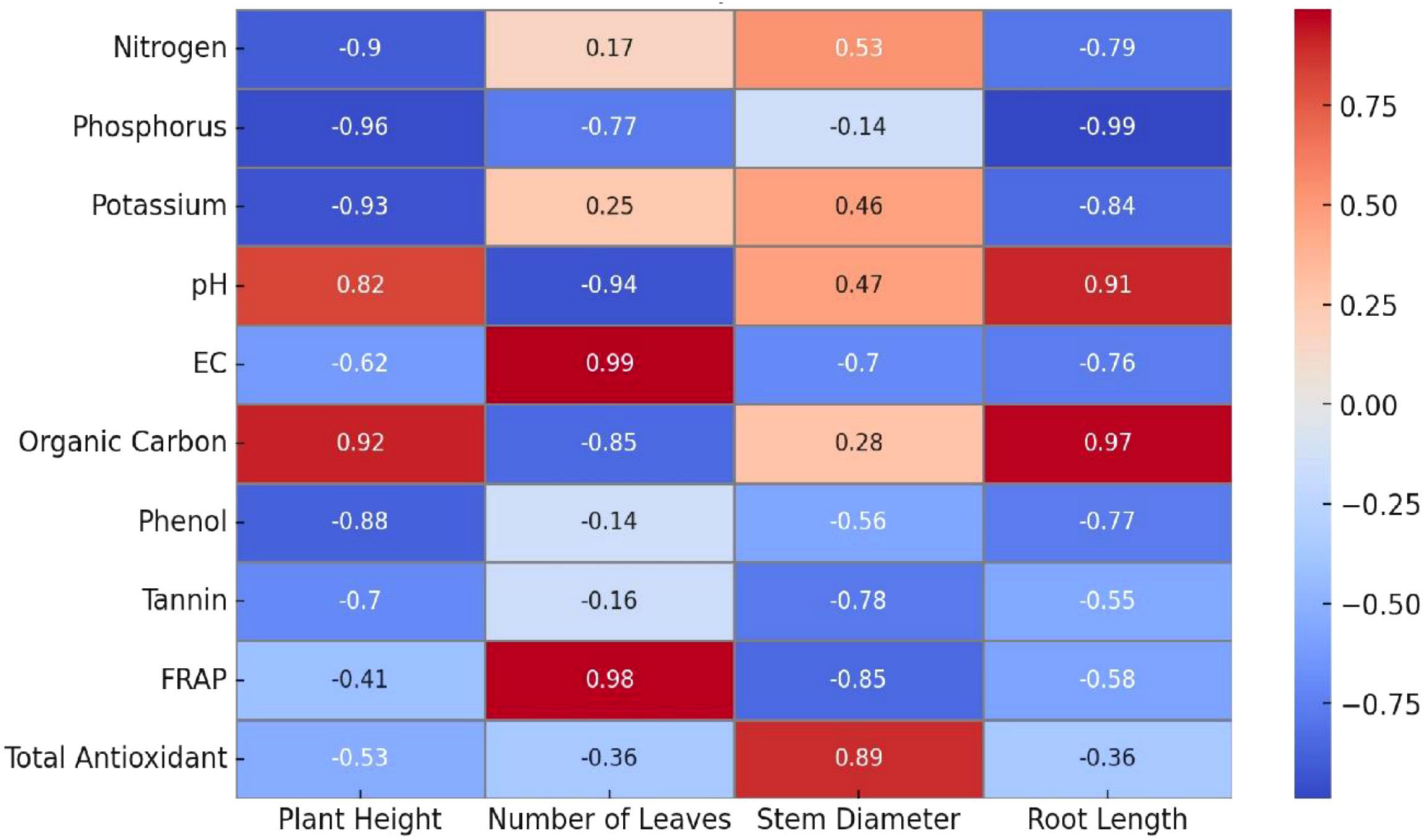
Heat map showing correlation patterns between soil physico-chemical properties, nodule biochemical traits, and growth parameters of *P. pinnata* seedlings, illustrating positive and negative associations influencing plant performance across study sites.

The results reveal distinct patterns of association indicating how soil fertility and biochemical factors influence plant performance. Nitrogen content exhibited a strong negative correlation with plant height (–0.90), implying that excessive nitrogen availability may inversely affect vertical growth. Similarly, phenol concentration was negatively correlated with plant height (–0.88), suggesting that higher phenolic accumulation could be associated with stress responses rather than vigorous growth. Phosphorus showed negative correlations with both the number of leaves (–0.77) and root length (–0.99), indicating that higher phosphorus availability may not necessarily enhance these growth attributes. Potassium also exhibited a strong negative relationship with root length (–0.84), reinforcing this trend. In contrast, EC was positively and strongly correlated with the number of leaves (0.99), suggesting that moderate ionic activity in soil favors leaf development. Organic carbon and pH demonstrated positive associations with root length (0.97 and 0.91, respectively), implying improved root growth under favorable organic and neutral to slightly alkaline conditions. Meanwhile, antioxidant parameters such as FRAP and total antioxidant content showed moderate to weak correlations with growth variables, reflecting their independent physiological roles. Overall, the correlation analysis highlights that while certain soil nutrients exert negative effects on seedling growth traits, parameters like EC, pH, and organic carbon positively contribute to root and leaf development in *P. pinnata* (**Figure 7**).

Multiple linear regression analysis further quantified the influence of soil and biochemical parameters on the growth of *P. pinnata* seedlings. For plant height, nitrogen, potassium, pH, organic carbon, tannin, and total antioxidant content contributed positively, while phosphorus, phenol, and electrical conductivity had negative effects, with the model explaining 85% of the variability (R² = 0.85): Plant Height (cm) = 0.175X_1_ − 2.127X_2_ + 0.217X_3_ + 1.059X_4_ − 0.601X_5_ + 0.392X_6_ − 0.738X_7_ + 0.412X_8_ − 0.081X_9_ + 0.056X_10_ + 12.438. The number of leaves was primarily negatively influenced by phosphorus and organic carbon, while nitrogen, potassium, pH, electrical conductivity, tannin, and total antioxidant content contributed slightly positively, with moderate predictability (R² = 0.55): Number of Leaves (No.) = 0.080X_1_ − 0.776X_2_ + 0.114X_3_ + 0.926X_4_ + 0.456X_5_ − 0.178X_6_ − 0.092X_7_ + 0.057X_8_ + 0.012X_9_ − 0.024X_10_ + 7.592. Stem diameter was less sensitive to soil and biochemical parameters, showing minor positive contributions from nitrogen, potassium, pH, and organic carbon, and a slight negative effect from phosphorus, electrical conductivity, and phenol, with an R² of 0.75: Stem Diameter (mm) = 0.001X_1_ − 0.016X_2_ + 0.003X_3_ + 0.008X_4_ − 0.005X_5_ + 0.002X_6_ − 0.001X_7_ + 0.000X_8_ − 0.000X_9_ + 0.000X_10_ + 0.298. Root length was strongly influenced by both promotive and inhibitory factors. Nitrogen, potassium, pH, organic carbon, tannin, and total antioxidant content positively affected root elongation, while phosphorus, phenol, and electrical conductivity showed negative effects, with an R² of 0.79: Root Length (cm) = 0.096X_1_ − 0.749X_2_ + 0.103X_3_ + 0.512X_4_ − 0.314X_5_ + 0.221X_6_ − 0.451X_7_ + 0.206X_8_ − 0.033X_9_ + 0.028X_10_ + 15.876.

Furthermore, Stepwise regression analysis further refined these relationships by selecting only the most influential variables. For plant height, nitrogen, pH, organic carbon, and tannin were positive contributors, while phosphorus and phenol were negative, with the model explaining 61% of variability (R² = 0.61): Plant Height (cm) = 0.168X_1_ − 1.954X_2_ + 0.978X_4_ + 0.347X_6_ − 0.652X_7_ + 0.298X_8_ + 11.872. The number of leaves was negatively affected by phosphorus, with minor positive contributions from nitrogen, pH, and tannin, reflecting moderate predictability (R² = 0.43): Number of Leaves (No.) = 0.076X_1_ − 0.612X_2_ + 0.802X_4_ + 0.058X_8_ + 7.145. Stem diameter showed small positive effects from potassium, pH, and organic carbon, with a slight negative influence from electrical conductivity (R² = 0.66): Stem Diameter (mm) = 0.002X_3_ + 0.007X_4_ + 0.001X_6_ − 0.004X_5_ + 0.283. Root length was influenced positively by nitrogen, potassium, pH, organic carbon, and tannin, while phosphorus and phenol had inhibitory effects, with an R² of 0.71: Root Length (cm) = 0.085X_1_ − 0.625X_2_ + 0.489X_4_ + 0.208X_6_ + 0.184X_8_ − 0.395X_7_ + 15.425. Where: X_1_ = Nitrogen, X_2_ = Phosphorus, X_3_ = Potassium, X_4_ = pH, X_5_ = Electrical Conductivity, X_6_ = Organic Carbon, X_7_ = Phenol, X_8_ = Tannin, X_9_ = [other variable if measured], X_10_ = Total Antioxidant Content. These results highlight that soil nutrient status and biochemical composition of nodules collectively play crucial roles in determining the growth performance of *P. pinnata* seedlings.

### Principal component analysis, hierarchical cluster and its relevance in understanding multivariate relationships between soil nutrients, seedling growth, and root nodule biochemical traits

**Figure 8:**
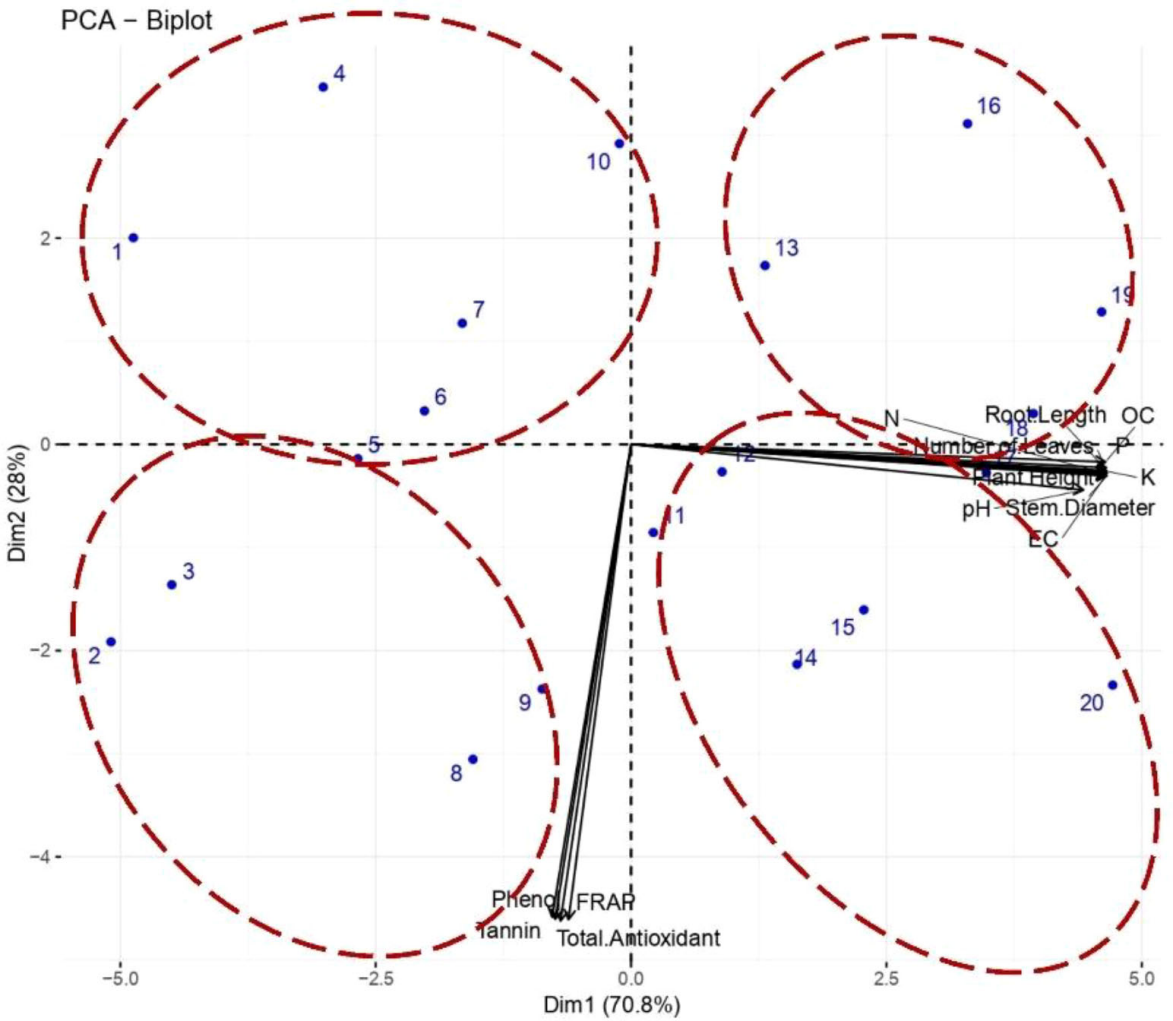
Principal Component Analysis (PCA) biplot showing the distribution of *P. pinnata* isolates (PP-01 to PP-20) based on soil physico-chemical properties, nodule biochemical traits, and growth parameters, illustrating the major factors contributing to total variance.

The PCA biplot of 20 P*. pinnata* isolates (PP-01 to PP-20) revealed clear grouping patterns based on their biochemical and growth attributes. Dim1 and Dim2 accounted for 70.8% and 28% of the total variance, respectively, indicating that most of the variation among isolates is captured by these two principal components. Isolates PP-01, PP-04, PP-05, PP-06, PP-07, and PP-10 clustered in the top-left quadrant, suggesting moderate to low phenol, tannin, FRAP, and total antioxidant activities (**Figure 8**). Isolates PP-02, PP-03, PP-08, PP-09, PP-11, PP-12, PP-14, PP-15, PP-17, and PP-18 were located closer to the bottom-left quadrant, indicating higher biochemical activities relative to the previous cluster. In contrast, PP-13, PP-16, PP-19, and PP-20 were positioned in the right quadrants, reflecting stronger contributions from growth parameters such as root length, plant height, stem diameter, and number of leaves. The biplot arrows indicate that phenol, tannin, FRAP, and total antioxidant activities are positively correlated with each other, while growth parameters (root length, plant height, stem diameter, number of leaves, EC, pH, OC, N, P, K) are closely aligned in another direction, suggesting that these two sets of traits contribute differently to the overall variability.

**Figure 9:**
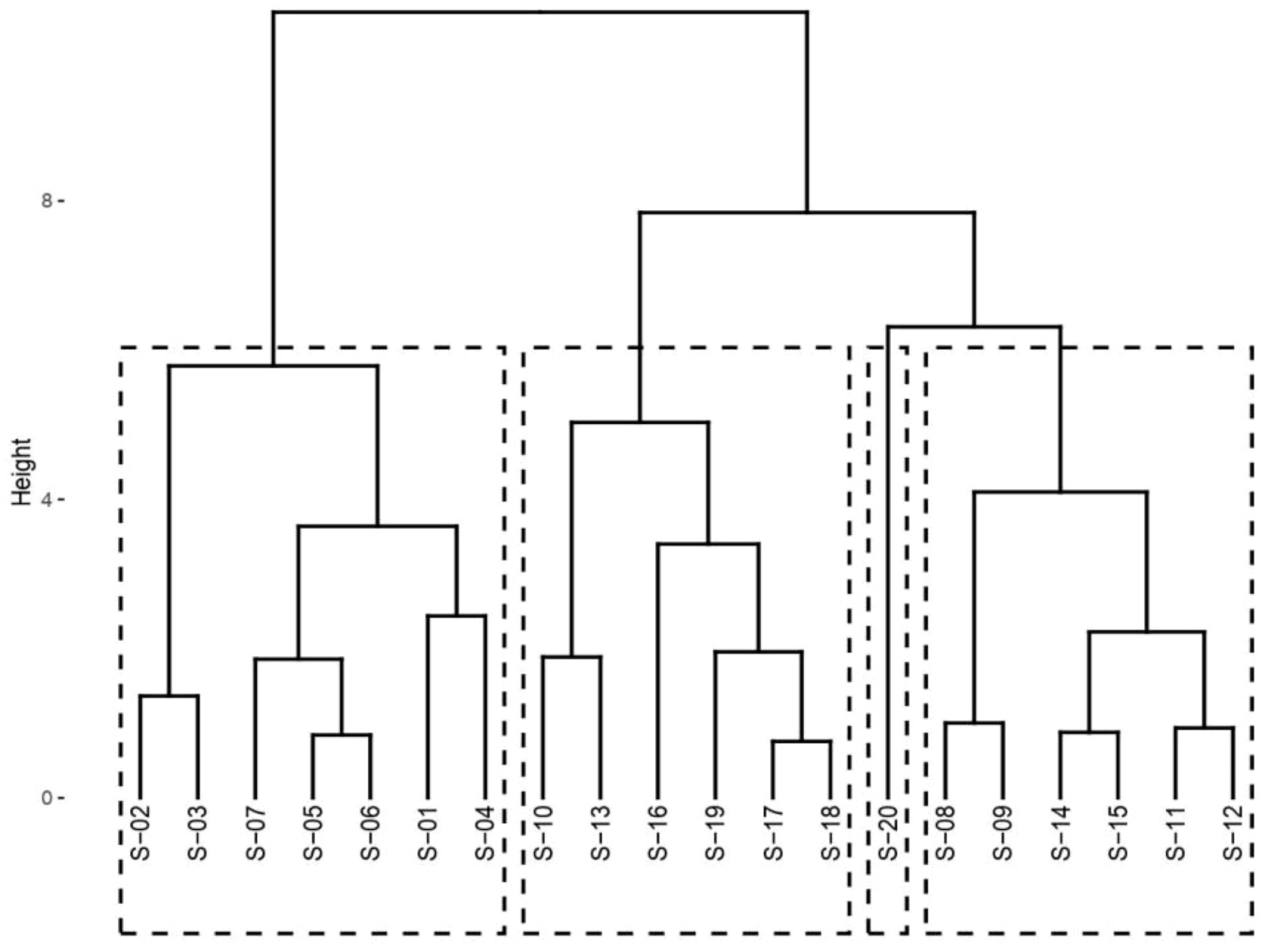
Hierarchical cluster analysis (HCA) dendrogram grouping 20 P*. pinnata* study sites into three clusters based on combined soil, biochemical, and growth attributes, highlighting spatial variability and site-specific associations in arid regions of western Rajasthan.

Furthermore, hierarchical cluster analysis grouped the twenty study sites of *P. pinnata* into three distinct clusters based on combined soil physicochemical and biochemical parameters along with plant growth traits. Cluster I comprised sites S01–S07, mainly representing locations from Barmer, Churu, and Jodhpur districts. These sites were characterized by relatively lower organic carbon and nutrient (NPK) content, corresponding with moderate plant height and leaf number. Cluster II included sites S10, S13, S16, S17, and S19, primarily from Jhunjhunu and Sikar districts, which exhibited higher organic carbon, phenolic, and antioxidant contents, indicating a comparatively enriched soil biochemical profile that supported better growth performance. Cluster III encompassed sites S08, S09, S11, S12, S14, S15, S18, and S20, where soil pH and electrical conductivity were relatively higher, but nutrient availability and growth parameters were moderate to low (**Figure 9**). The clustering pattern suggests that edaphic variability, particularly in soil organic carbon, nitrogen, and phenolic compounds, plays a critical role in influencing the growth response of *P. pinnata* across different arid and semi-arid locations.

## Discussion

The present study revealed pronounced spatial variability in the nutrient content and physico-chemical properties of rhizospheric soils of *P. pinnata* across the 20 sampling locations, highlighting the heterogeneity of arid region soils in western Rajasthan. The observed variations in nitrogen, phosphorus, potassium, pH, electrical conductivity, and organic carbon indicate the combined influence of natural edaphic factors, vegetation cover, and localized soil–plant interactions. Similar variability in rhizospheric soil fertility has been reported by Singh et al. (2020) and Talha Bin Yousaf et al. (2022) in leguminous tree species growing under arid and semi-arid conditions. The relatively higher nutrient concentrations, particularly in sites S-16 to S-20, suggest an enhanced rhizosphere effect, likely due to the active litter deposition and microbial activity associated with *P. pinnata* roots. Comparable findings were reported by Kumar M. et al. (2025), who observed improved nitrogen and organic carbon levels in the rhizospheric zones of leguminous trees in degraded arid soils (Dhaliwal et al., 2023). The elevated soil pH and EC recorded in the present study agree with the findings of Enagbonma et al. (2023), who noted that the rhizospheres of desert tree species often reflect the calcareous and alkaline nature of Thar desert soils (Abdelaal et al., 2021). Organic carbon levels were generally low, as expected in arid ecosystems with limited organic inputs and rapid decomposition under high temperatures (Gupta et al., 2019). Nevertheless, the higher OC values recorded at S-16 to S-20 indicate localized enrichment, likely influenced by litterfall and root exudates of *P. pinnata*, consistent with the observations of Moharana et al. (2021) in the rhizospheric soils of arid-zone legumes.

Furthermore, the present study demonstrated significant variability in the growth performance of *P. pinnata* seedlings, as well as in the morphological and biochemical characteristics of their root nodules, across different rhizospheric soil sites in the arid regions of western Rajasthan. The superior growth of seedlings in sites S-16 to S-20 compared to those from S-01 to S-05 highlights the strong influence of soil fertility and rhizospheric microbial interactions on early seedling development. Similar site-dependent growth responses of leguminous tree seedlings have been reported by Sahoo et al. (2021), who attributed these differences to variations in nutrient availability and the presence of effective *Rhizobium* populations in the rhizosphere. The morphological diversity of nodules observed in the present study ranging from spherical to elongated and irregular shapes is consistent with findings by Khandelwal and Vyas (2024) on native *Rhizobium* isolates from arid legumes, where spherical nodules were reported as predominant and associated with higher nitrogen fixation efficiency. The pink to pinkish-red coloration of nodule interiors recorded in all isolates confirms their effectiveness in active symbiotic nitrogen fixation, aligning with the results of Rajarajan et al. (2022), who reported similar pigmentation in nodules of leguminous species as a reliable indicator of leghemoglobin activity. The biochemical characterization of nodules revealed substantial differences in phenolic, tannin, FRAP, and antioxidant contents among isolates, with certain isolates (PP-02, PP-08) consistently showing higher levels. These findings are in agreement with those of Shoaib et al. (2021), who reported that phenolic and antioxidant compounds in nodules enhance the plant’s ability to tolerate abiotic stress and improve the overall symbiotic efficiency. The higher FRAP and total antioxidant contents recorded in several isolates also support earlier observations by Maya (2022) and Marriboina et al. (2022), who emphasized the role of antioxidant activity in promoting nodulation efficiency and maintaining nodule integrity under arid conditions. Plant phenolics and tannins are highlighted for their antioxidant and protective roles, mitigating oxidative damage caused by abiotic stressors such as high temperature, salinity, and water deficit (Maya, 2022). Elevated FRAP and total antioxidant levels are now linked to enhanced nodule longevity, structural integrity, and overall symbiotic efficiency by protecting nitrogen-fixing bacteria from reactive oxygen species (Rajarajan et al., 2022; Marriboina et al., 2022). This explanation also underscores how these compounds contribute to the efficiency of nitrogen fixation (Shoaib et al., 2021) and the functional performance of the *Rhizobium-Pongamia* symbiosis in harsh arid ecosystems, thereby justifying their measurement in the present study.

Furthermore, the present study highlights the significant phenotypic, biochemical, and functional diversity among RNB isolates associated with *P. pinnata* in arid soils. The isolates exhibited notable variations in colony morphology, salt tolerance, pH adaptability, and BTB reactions, reflecting their ecological plasticity in coping with the heterogeneous soil conditions typical of semi-arid and arid ecosystems. These findings align with the reports of Khandelwal and Vyas (2024) who documented that rhizobial populations inhabiting arid and saline soils often display wide phenotypic variability, particularly in salt tolerance and pH adaptability, which is crucial for maintaining effective symbiosis under stressful edaphic conditions (Maya, 2022).

The intrinsic antibiotic resistance assays revealed substantial heterogeneity in the response of isolates to commonly tested antibiotics (Liu et al., 2022), a trait often linked to the natural soil environment and prior exposure to abiotic stresses (Mir et al., 2020). Similar observations were reported by Sadowsky and Graham (2008), who noted that native rhizobia often carry varying degrees of intrinsic resistance to antibiotics, which can contribute to their persistence and competitive advantage in the rhizosphere. The resistance patterns detected in several isolates in the present study may also reflect their potential resilience against anthropogenic disturbances such as agricultural chemical inputs (Abera et al., 2015).

Biochemical characterization further underscored the functional diversity among isolates, with widespread nitrate reduction and oxidase activity but limited catalase and amylase expression (Krishnasamy and Obbineni, 2025). Comparable results were obtained by Agustiani et al. (2023), who emphasized that differences in enzymatic profiles can influence rhizobial competitiveness and symbiotic performance. The absence of gelatin hydrolysis in all isolates is consistent with reports from Chiranjeevi (2020), suggesting that extracellular protease production is not a ubiquitous trait in rhizobial species associated with leguminous trees (Krishnasamy et al., 2024).

The plant growth-promoting (PGP) activities revealed IAA production as a universal trait, which agrees with the findings of Kashyap et al. (2019) who demonstrated that auxin production is a common mechanism by which rhizobia stimulate host root elongation and seedling vigor. However, the limited occurrence of phosphate solubilization, ammonia production, chitinase, and pectinase activities indicates that these functions are strain-specific and not characteristic of all *P. pinnata*-associated rhizobia. Similar patterns were documented by Tsegaye et al. (2019), who reported that tree-associated rhizobia often exhibit narrow PGP profiles due to ecological specialization and selective pressures of their host plant environments. The combined phenotypic, biochemical, and PGP traits identified in the present study suggest that certain isolates, such as those demonstrating higher salt tolerance, antibiotic resistance, and superior enzymatic activities, may serve as promising candidates for bio-inoculant development to support *P. pinnata* cultivation in nutrient-poor and saline soils (Vashistha et al., 2024). These findings, corroborated by earlier studies on stress-tolerant rhizobia, underscore the potential of exploiting native microbial diversity for sustainable afforestation and biofuel production in arid regions (Shi et al., 2022).

The findings of the present study demonstrate a strong interplay between soil nutrient status, biochemical composition of root nodules, and the growth response of *P. pinnata* seedlings. Correlation analysis revealed that certain soil nutrients and biochemical parameters were closely associated with specific growth traits. Nitrogen exhibited a significant negative correlation with plant height (-0.90*), indicating that higher soil nitrogen levels did not necessarily enhance vertical growth, possibly due to nutrient imbalances or excessive nitrogen leading to altered allocation of assimilates. Similarly, phenol content in the root nodules showed a strong negative correlation with plant height (-0.88**), suggesting that elevated phenolic compounds may have inhibitory effects on elongation growth. These results align with previous studies highlighting the complex interactions between soil nutrients, biochemical compounds, and plant growth. Chimdi et al. (2022) observed that soil physicochemical properties significantly affect plant growth, including pollen performance, suggesting that nutrient levels can influence various growth parameters. Similarly, Hasan (2018) reported that certain rhizobial isolates with high nitrogen-fixing capacity promoted *P. pinnata* growth by increasing nitrogen content, emphasizing the role of nitrogen availability in plant development.

Phosphorus showed a significant negative correlation with the number of leaves (−0.77*) and root length (−0.99*), while potassium was strongly negatively correlated with root length (−0.84**), suggesting that excess nutrients may limit leaf and root development. EC was positively correlated with the number of leaves (0.99**), indicating that moderate salinity might enhance leaf proliferation. Multiple regression analysis highlighted phosphorus and phenol as major negative contributors to plant height and root length, whereas nitrogen, potassium, pH, organic carbon, tannin, and EC had positive effects. The R² values indicated strong predictive relationships for plant height (0.85) and root length (0.79), with moderate effects on stem diameter (0.75) and number of leaves (0.55). Furthermore, Shen et al. (2023) demonstrated that long-term phytoremediation using symbiotic legumes could reshape soil microbial communities (Yu et al., 2021), indicating that plant growth can influence and be influenced by soil microbial dynamics (Kumar N. et al., 2025). Degani et al. (2022) reviewed multiple applications of *P. pinnata*, noting its varying levels of tolerance to drought, salinity, and heavy metals in soils, which could affect its growth response under different environmental conditions.

These results indicate that both soil nutrient composition and nodule biochemistry substantially affect the growth dynamics of *P. pinnata* seedlings. Excessive concentrations of certain nutrients, particularly phosphorus and potassium, together with high phenolic content in nodules, may hinder optimal growth. The findings underscore the importance of maintaining balanced soil fertility and managing biochemical responses in root nodules to achieve improved seedling vigor and sustainable plantation establishment in arid regions.

## Conclusion

The present study provides a comprehensive assessment of soil nutrient status, rhizobial diversity, and their combined influence on the growth and vigor of *P. pinnata* seedlings under arid conditions of western Rajasthan. Soils from different sites exhibited substantial variability in fertility parameters such as nitrogen, phosphorus, potassium, pH, EC, and organic carbon, which in turn influenced seedling growth, root development, and nodule formation. Seedlings grown in nutrient-enriched soils (S-16 to S-20) showed superior growth traits, highlighting the critical role of rhizospheric soil quality in successful seedling establishment. Morphological and biochemical characterization of root nodules revealed diversity in size, shape, and pigmentation, reflecting active nitrogen fixation. Among the twenty isolates studied, PP-02 (Sindhari, S-02, Barmer), PP-05 (Tal Chappar, S-05, Churu), PP-08 (Balesar, S-08, Jodhpur), PP-11 (Pilani, S-11, Jhunjhunu), PP-18 (Laxmangarh, S-18, Sikar), and PP-20 (Reengus, S-20, Sikar) demonstrated superior biochemical traits, including higher phenolic content, FRAP, and antioxidant capacity, along with tolerance to salinity (up to 3% NaCl) and a wide pH range (5–11). These isolates also consistently exhibited plant growth-promoting traits, particularly IAA production, although other traits such as phosphate solubilization, ammonia secretion, and chitinase/pectinase activity were strain-specific. Their combination of stress tolerance and growth-promoting capabilities makes them promising candidates for bioinoculant development aimed at improving *P. pinnata* seedling establishment and resilience in arid soils. Correlation and regression analyses revealed strong linkages between soil fertility, nodule biochemistry, and seedling growth, suggesting that phosphorus and phenolic compounds can sometimes negatively influence growth, whereas nitrogen, potassium, and EC support optimal seedling development. These findings provide actionable insights for forestry and restoration programs, guiding the selection of nutrient-rich soils and compatible rhizobial inoculants to enhance plantation success.

Future research should focus on the long-term field validation of superior rhizobial isolates across multiple sites to evaluate their performance under variable climatic and edaphic conditions. In parallel, genomic and metabolomic profiling of these elite isolates can identify functional genes associated with nitrogen fixation, stress tolerance, and plant growth promotion, supporting the rational selection of robust bioinoculants. Additionally, the development of effective inoculant formulations for seed coating, soil amendment, or nursery application is essential to translate laboratory findings into practical, scalable field practices. In conclusion, this study not only enhances understanding of soil–microbe–plant interactions in arid ecosystems but also identifies elite rhizobial isolates with practical potential for sustainable afforestation, climate-resilient plantations, and biofuel-oriented cultivation of *P. pinnata* in water-limited, nutrient-poor environments.

## Data Availability

The original contributions presented in the study are included in the article/**Supplementary Material**. Further inquiries can be directed to the corresponding authors.

## References

[B1] Abdelaal K. AlKahtani M. Attia K. Hafez Y. Király L. Künstler A. (2021). The role of plant growth-promoting bacteria in alleviating the adverse effects of drought on plants. Biology 10, 520. doi: 10.3390/biology10060520, PMID: 34207963 PMC8230635

[B2] Abera T. Semu E. Debele T. Wegary D. Kim H. (2015). Determination soil rhizobium populations, intrinsic antibiotic resistance, nodulation and seed yield of faba bean and soybean in Western Ethiopia. World J. Agric. Sci. 11, 311–324. doi: 10.5829/idosi.wjas.2015.11.5.1869

[B3] Aguilar C. H. Pires D. Cortaga C. Peja R. Jr. Cruz M. A. Langres J. . (2025). Harnessing legume productivity in tropical farming systems by addressing challenges posed by legume diseases. Nitrogen 6, 65. doi: 10.3390/nitrogen6030065

[B4] Agustiani R. D. Rahmani N. Ekowati N. (2023). Isolation and characterization of rhizospheric bacteria associated with canna plant: for production of maltooligosaccharide amylase (Jakarta, Indonesia: Asadel Publisher).

[B5] Ameer S. Ibrahim H. Kulsoom F. N. U. Ameer G. Sher M. (2024). Real-time detection and measurements of nitrogen, phosphorous & potassium from soil samples: a comprehensive review. J. Soils Sediments 24, 2565–2583. doi: 10.1007/s11368-024-03827-5

[B6] Amorim E. L. Nascimento J. E. Monteiro J. M. Peixoto Sobrinho T. J. Araújo T. A. Albuquerque U. P. (2008). A simple and accurate procedure for the determination of tannin and flavonoid levels and some applications in ethnobotany and ethnopharmacology. Funct. Ecosyst. Commun. 2, 88–94.

[B7] Benzie I. F. Strain J. J. (1999). Ferric reducing/antioxidant power assay: direct measure of total antioxidant activity of biological fluids and modified version for simultaneous measurement of total antioxidant power and ascorbic acid concentration. Methods enzymology 299, 15–27. doi: 10.1016/S0076-6879(99)99005-5, PMID: 9916193

[B8] Bhati K. T. Kumar S. Haileslassie A. Whitbread A. M. (2017). Assessment of agricultural technologies for Dryland systems in South Asia: a case study of Western Rajasthan, India. 68.

[B9] Bhoi T. K. Samal I. Saraswat A. Hombegowda H. C. Samal S. K. Dash A. K. . (2024). “ Biochar as a soil amendment: Effects on microbial communities and soil health,” in Biochar production for green economy (London, United Kingdom: Academic Press), 137–159.

[B10] Bhusal A. Muriana P. M. (2021). Isolation and characterization of nitrate reducing bacteria for conversion of vegetable-derived nitrate to ‘natural nitrite’. Appl. Microbiol. 1, 11–23. doi: 10.3390/applmicrobiol1010002

[B11] Cappuccino J. G. Sherman N. (2007). Microbiology, A laboratory manual. 7th ed (California, USA: The Benjamin/Cummings Publishing Co.).

[B12] Cappuccino J. G. Sherman N. (2008). *Microbiology: a laboratory manual* (Vol. 9) (San Francisco, CA, USA: Pearson/Benjamin Cummings).

[B13] Chimdi A. Negasa D. Chala G. (2022). Effects of rhizobium inoculation and P fertilizer levels on selected soil properties, yield, and yield components of faba bean (Vicia faba L.): The case of Abuna Gindeberat, west Shewa Zone, Ethiopia. Appl. Environ. Soil Sci. 2022, 3635989. doi: 10.1155/2022/3635989

[B14] Chiranjeevi N. (2020). *Investigations into antagonistic basis of root endophytic bacteria in the control of chickpea dry root rot pathogen (Rhizoctonia bataticola (Taub.) Butler)*. Guntur, Andhra Pradesh, India: ACHARYA NG RANGA AGRICULTURAL UNIVERSITY.

[B15] Degani E. Prasad M. V. R. Paradkar A. Pena R. Soltangheisi A. Ullah I. . (2022). A critical review of Pongamia pinnata multiple applications: from land remediation and carbon sequestration to socioeconomic benefits. J. Environ. Manage. 324, 116297. doi: 10.1016/j.jenvman.2022.116297, PMID: 36174475

[B16] Dhaliwal S. S. Sharma V. Shukla A. K. Kaur J. Gupta R. K. Verma V. . (2023). Interactive effect of land use systems on depth-wise soil properties and micronutrients minerals in North-Western, India. Current science 116 (1), 112–116. doi: 10.1016/j.heliyon.2023.e13591, PMID: 36865444 PMC9970903

[B17] Enagbonma B. J. Fadiji A. E. Ayangbenro A. S. Babalola O. O. (2023). Communication between plants and rhizosphere microbiome: exploring the root microbiome for sustainable agriculture. Microorganisms 11, 2003. doi: 10.3390/microorganisms11082003, PMID: 37630562 PMC10458600

[B18] Fernandes Júnior P. I. Lima A. A. D. Passos S. R. Gava C. A. T. Oliveira P. J. D. Rumjanek N. G. . (2012). Phenotypic diversity and amylolytic activity of fast growing rhizobia from pigeonpea [*Cajanus cajan* (L.) Millsp. Braz. J. Microbiol. 43, 1604–1612. doi: 10.1590/S1517-83822012000400045, PMID: 24031992 PMC3769046

[B19] Gebreegziabher B. W. Dubale A. A. Adaramola M. S. Morken J. (2025). Advancing anaerobic digestion of biodiesel byproducts: A comprehensive review. Bioenergy Res. 18, 15. doi: 10.1007/s12155-025-10820-4

[B20] Giordano M. Petropoulos S. A. Rouphael Y. (2021). The fate of nitrogen from soil to plants: Influence of agricultural practices in modern agriculture. Agriculture 11, 944. doi: 10.3390/agriculture11100944

[B21] Gordon S. A. Weber R. P. (1951). Colorimetric estimation of indoleacetic acid. Plant Physiol. 26, 192–195. doi: 10.1104/pp.26.1.192, PMID: 16654351 PMC437633

[B22] Goyal R. K. Habtewold J. Z. (2023). Evaluation of legume–rhizobial symbiotic interactions beyond nitrogen fixation that help the host survival and diversification in hostile environments. Microorganisms 11, 1454. doi: 10.3390/microorganisms11061454, PMID: 37374957 PMC10302611

[B23] Goyal R. K. Schmidt M. A. Hynes M. F. (2021). Molecular biology in the improvement of biological nitrogen fixation by rhizobia and extending the scope to cereals. Microorganisms 9, 125. doi: 10.3390/microorganisms9010125, PMID: 33430332 PMC7825764

[B24] Goyal K. Singh N. Jindal S. Kaur R. Goyal A. Awasthi R. (2022). Kjeldahl method. Advanced techniques analytical Chem. 1, 105. doi: 10.2174/97898150502331220101

[B25] Graham P. H. (2008). Ecology of the root-nodule bacteria of legumes. In Dilworth M. J. James E. K. Sprent J. I. Newton W. E. (Eds.), Nitrogen-fixing leguminous symbioses (pp. 23–58). Springer Netherlands. doi: 10.1007/978-1-4020-3548-2_2

[B26] Hasan M. (2018). Effect of rhizobium inoculation with phosphorus and nitrogen fertilizer on physico-chemical properties of the groundnut soil. Environ. Ecosystem Sci. 2, 04–06. doi: 10.26480/ees.01.2018.04.06

[B27] Isokar S. Potdukhe S. Ingle R. Pudake S. P. (2024). Characterization of Mesorhizobium ciceri isolates from chickpea root nodules: A biochemical approach. Int. J. Advanced Biochem. Res. 8, 753–759. doi: 10.33545/26174693.2024.v8.i4i.1042

[B28] Kasana R. C. Salwan R. Dhar H. Dutt S. Gulati A. (2008). A rapid and easy method for the detection of microbial cellulases on agar plates using Gram’s Iodine. Curr. Microbiol. 57, 503–507. doi: 10.1007/s00284-008-9276-8, PMID: 18810533

[B29] Kashyap A. S. Tetorya M. Kesharwani A. K. Singh D. (2019). MOLECULAR AND PHYSIOLOGICAL CHARACTERIZATION OF PLANT GROWTH PROMOTING RHIZOBACTERIA: METHODS AND PROTOCOLS. Pharmacognosy Nutr. 79, 79–104.

[B30] Khandelwal M. Vyas P. S. (2024). Cultural and biochemical characteristics of rhizobium present in nodule of pongamia pinnata (l.) pierre. Indian J. Advanced Bot. (IJAB) 4, 32–36. doi: 10.54105/ijab.B1036.04021024

[B31] Khare N. Khare P. Singh S. (2025). “ Molecular and physiological concepts: Macronutrients in crop plant growth and development,” in Agricultural crop improvement (Boca Raton, FL, USA: CRC Press), 148–164).

[B32] Krishnasamy R. Natesh R. Obbineni J. M. (2024). Efficient ROS scavenging improves the growth and yield in black gram (Vigna mungo (L.) Hepper) after seed priming and treatment using biosynthesized silver nanoparticles with Pongamia pinnata (L.) Pierre leaf extract. J. Plant Growth Regul. 43, 2422–2438. doi: 10.1007/s00344-024-11276-0

[B33] Krishnasamy R. Obbineni J. M. (2025). Phytopriming of Vigna mungo (L.) Hepper (Blackgram) Seeds using Aqueous Extracts of Moringa oleifera Lam, Gliricidia sepium (Jacq.) Walp and Pongamia pinnata (L.) Pierre Improves Seedling Emergence, Growth and Yield. Indian J. Agric. Res. 59, 2422–2438. doi: 10.18805/ijare.a-6334

[B34] Kumar M. Mitra S. Mazumdar S. P. Verma B. C. Pramanick B. (2025). System productivity, soil carbon and nitrogen sequestration of intensive rice-based cropping systems can be improved through legume crop inclusion with appropriate fertilizer application and crop residues incorporation in the eastern Indo-Gangatic plain. Plant Soil 507, 25–46. doi: 10.1007/s11104-023-06415-7

[B35] Kumar N. Pathak L. Shah K. (2025). Exploring the contribution of non-edible plants in phytoremediation and biofuel production in India. Environ. Sustainability, 1–14. doi: 10.1007/s42398-025-00343-1

[B36] Kundu A. Kundu C. K. Dey P. Rana S. Majumder J. Bera A. . (2024). Management of soil cover and tillage regimes in upland rice-sweet corn systems for better system performance, energy use and carbon footprints. Heliyon 10, e26524. doi: 10.1016/j.heliyon.2024.e26524, PMID: 38420378 PMC10900818

[B37] Liu B. Zhang D. Pan X. (2022). Nodules of wild legumes as unique natural hotspots of antibiotic resistance genes. Sci. Total Environ. 839, 156036. doi: 10.1016/j.scitotenv.2022.156036, PMID: 35597353

[B38] Marriboina S. Sekhar K. M. Subramanyam R. Reddy A. R. (2022). Physiological, biochemical, and root proteome networks revealed new insights into salt tolerance mechanisms in Pongamia pinnata (L.) Pierre. Front. Plant Sci. 12, 771992. doi: 10.3389/fpls.2021.771992, PMID: 35140728 PMC8818674

[B39] Maya R. (2022). *Studies on biochemical and molecular characterization of isolated nodule-associated bacteria from Mimosa pudica L. and their plant growth promoting activities on Vigna radiata L.(Wilczek)*. [Master’s/MPhil/Ph.D. thesis, University of Calicut]. Malappuram, Kerala, India Chiranjeevi: Department of Botany, University of Calicut.

[B40] Mehata D. K. Kattel I. Sapkota P. Ghimire N. P. Mehta R. K. (2023). Biofertilizers: A sustainable strategy for organic farming that would increase crop production and soil health. Plant Physiol. Soil Chem. 3, 49–53. doi: 10.26480/ppsc.02.2023.49.53

[B41] Mendoza-Suárez M. Andersen S. U. Poole P. S. Sánchez-Cañizares C. (2021). Competition, nodule occupancy, and persistence of inoculant strains: key factors in the rhizobium-legume symbioses. Front. Plant Sci. 12, 690567. doi: 10.3389/fpls.2021.690567, PMID: 34489993 PMC8416774

[B42] Mir M. I. Nagabhushanam B. Quadriya H. U. M. E. R. A. Kumar B. K. Hameeda B. (2020). Morphological, biochemical and intrinsic antibiotic resistance of rhizobia isolated from root and stem nodules of various leguminous plants. Plant Cell Biotechnol. Mol. Biol. 21, 126–138.

[B43] Moharana P. C. Jena R. K. Kumar N. Singh R. S. Rao S. S. (2021). Assessment of soil organic and inorganic carbon stock at different soil depths after conversion of desert into arable land in the hot arid regions of India. Carbon Manage. 12, 153–165. doi: 10.1080/17583004.2021.1893128

[B44] Mrunalini K. Behera B. Chandana P. Patnaik G. P. Modi R. U. Saraswat A. . (2022). “ Legumes to reduce ecological footprints for climate-smart cropping systems,” in Advances in legumes for sustainable intensification (London, United Kingdom: Academic Press), 403–420.

[B45] Mukherjee R. Sen S. (2021). Role of biological nitrogen fixation (BNF) in sustainable agriculture: A Review. Int. J. Adv. Life Sci. Res. 4, 01–07. doi: 10.31632/ijalsr.2021.v04i03.001

[B46] Mulla A. A. Shah A. P. (2025). Production and engine performance analysis of biodiesel from Pongamia pinnata using PTSA and CaO nanocatalysts. Biomass Bioenergy 195, 107714. doi: 10.1016/j.biombioe.2025.107714

[B47] Nag P. Shriti S. Das S. (2020). Microbiological strategies for enhancing biological nitrogen fixation in nonlegumes. J. Appl. Microbiol. 129, 186–198. doi: 10.1111/jam.14557, PMID: 31858682

[B48] Naorem A. Jayaraman S. Dang Y. P. Dalal R. C. Sinha N. K. Rao C. S. . (2023). Soil constraints in an arid environment—Challenges, prospects, and implications. Agronomy 13, 220. doi: 10.3390/agronomy13010220

[B49] Nautiyal C. S. (1999). An efficient microbiological growth medium for screening phosphate solubilizing microorganisms. FEMS Microbiol. Lett. 170, pp.265–pp.270. doi: 10.1111/j.1574-6968.1999.tb13383.x, PMID: 9919677

[B50] Nemenzo P. (2016). Nodulation and symbiotic nitrogen fixation in the biofuel legume tree Pongamia pinnata. Atlas J. Biol. 2016, 274–291. doi: 10.5147/ajb.2016.0141

[B51] Nohwar N. Khandare R. V. Desai N. S. (2019). Isolation and characterization of salinity tolerant nitrogen fixing bacteria from Sesbania sesban (L) root nodules. Biocatalysis Agric. Biotechnol. 21, 101325. doi: 10.1016/j.bcab.2019.101325

[B52] Ortíz J. Sanhueza C. Romero-Munar A. Hidalgo-Castellanos J. Castro C. Bascuñán-Godoy L. . (2020). *In vivo* metabolic regulation of alternative oxidase under nutrient deficiency—interaction with arbuscular mycorrhizal fungi and rhizobium bacteria. Int. J. Mol. Sci. 21, 4201. doi: 10.3390/ijms21124201, PMID: 32545597 PMC7349880

[B53] Park S. Shin Y. Kim J. M. Kim M. S. Jung S. (2024). Rhizobial oxidized 3-hydroxylbutanoyl glycan-based gelatin hydrogels with enhanced physiochemical properties for pH-responsive drug delivery. Int. J. Biol. Macromolecules 264, 130538. doi: 10.1016/j.ijbiomac.2024.130538, PMID: 38432278

[B54] Patil J. R. Mhatre K. J. Yadav K. Yadav L. S. Srivastava S. Nikalje G. C. (2024). Flavonoids in plant-environment interactions and stress responses. Discover Plants 1, 68. doi: 10.1007/s44372-024-00063-6

[B55] Paudyal S. P. Kunwar B. Paudel N. Das B. D. (2021). Isolation and characterization of rhizobia from the root nodule of some cultivated legume crops. Eur. J. Biol. Res. 11, 294–306. doi: 10.5281/zenodo.4906255

[B56] Prieto P. Pineda M. Aguilar M. (1999). Spectrophotometric quantitation of antioxidant capacity through the formation of a phosphomolybdenum complex: specific application to the determination vitamin E. Anal. Biochem. 269, 337–341. doi: 10.1006/abio-.1999.4019, PMID: 10222007

[B57] Rajarajan K. Sakshi S. Taria S. Prathima P. T. Radhakrishna A. Anuragi H. . (2022). Whole plant response of Pongamia pinnata to drought stress tolerance revealed by morpho-physiological, biochemical and transcriptome analysis. Mol. Biol. Rep. 49, 9453–9463. doi: 10.1007/s11033-022-07808-0, PMID: 36057878

[B58] Ramanjaneyulu A. V. Chaitanya T. Raghu Rami Reddy P. Rajashekhar M. Handa A. K. Arunachalam A. . (2025). Monograph on “Pongamia”(Pongamia pinnata) Vol. 500 (Rajendranagar, Hyderabad, India: Professor Jayashankar Telangana Agricultural University (PJTAU), Rajendranagar, Hyderabad), 1–86.

[B59] Ronald M. A. James W. S. (2006) in 2nd ed (Boca Raton, Florida, United States: CRC Press).

[B60] Sahoo G. R. Swamy S. L. Mishra A. Thakur T. K. (2021). Effect of seed source, light, and nitrogen levels on biomass and nutrient allocation pattern in seedlings of Pongamia pinnata. Environ. Sci. pollut. Res. 28, 15005–15020. doi: 10.1007/s11356-020-11734-8, PMID: 33221992

[B61] Saritha M. Kumar S. Naorem A. K. Meena O. P. Meena K. K. Singh D. (2025). “ Fostering the future of drylands: balancing productivity and resilience through nature-based solutions,” in Land restoration through ecosystem-based approach: contexts from drylands of the global south ( Springer Nature Singapore, Singapore), 129–165.

[B62] Sharma S. Saraswat A. Khardia N. Chawla R. Ram S. Kumar R. . (2024). “ Soil-based implication approach for environmental nexus,” in Environmental nexus for resource management (Boca Raton, FL, USA: CRC Press), 67–80.

[B63] Sharma S. Singh Y. V. Saraswat A. Meena R. Khardia N. (2021). Soil quality assessment of different villages of Sanganer block in Jaipur district of Rajasthan (India). Environment & Ecology. 39 (4A), 1106–1113.

[B64] Shen T. Jin R. Yan J. Cheng X. Zeng L. Chen Q. . (2023). Study on diversity, nitrogen-fixing capacity, and heavy metal tolerance of culturable Pongamia pinnata rhizobia in the vanadium-titanium magnetite tailings. Front. Microbiol. 14, 1078333. doi: 10.3389/fmicb.2023.1078333, PMID: 37405163 PMC10315665

[B65] Shi P. Zhang J. Li X. Zhou L. Luo H. Wang L. . (2022). Multiple metabolic phenotypes as screening criteria are correlated with the plant growth-promoting ability of rhizobacterial isolates. Front. Microbiol. 12, 747982. doi: 10.3389/fmicb.2021.747982, PMID: 35069464 PMC8767003

[B66] Shihab M. A. Alkurtany E. S. (2023). “ Isolation and diagnosis of the root nodule bacteria associated with mung bean plant in gypsiferous soil and testing the promotional criterion of isolates,” in IOP Conference Series: Earth and Environmental Science, Vol. 1259. 012019 (Bristol, United Kingdom: IOP Publishing).

[B67] Shoaib M. Hussain S. Cheng X. Cui Y. Liu H. Chen Q. . (2021). Synergistic anti-oxidative effects of Pongamia pinnata against nickel mediated by Rhizobium pisi and Ochrobacterium pseudogrignonense. Ecotoxicology Environ. Saf. 217, 112244. doi: 10.1016/j.ecoenv.2021.112244, PMID: 33933891

[B68] Siddiqui A. R. Shahzad S. M. Ashraf M. Yasmeen T. Kausar R. Albasher G. . (2021). Development and characterization of efficient k-solubilizing rhizobacteria and mesorhizobial inoculants for chickpea. Sustainability 13 (18), 10240. doi: 10.3390/su131810240

[B69] Singh S. Bhoi T. K. Vyas V. (2023). “ Interceding microbial biofertilizers in agroforestry system for enhancing productivity,” in Plant growth promoting microorganisms of arid region ( Springer Nature Singapore, Singapore), 161–183.

[B70] Singh G. Nagora P. R. Haksar P. Kulshrestha S. Rani A. (2025). Assessing soil quality and biomass productivity under wastewater irrigation in the Indian arid region. Environ. Qual. Manage. 34, e70051. doi: 10.1002/tqem.70051

[B71] Singh A. Pant K. S. Prakash P. (2020). Effect of multipurpose tree species on soil Physico-chemical properties and available nutrients in subtropical region of Himachal Pradesh. J. Pharmacognosy Phytochem. 9, 935–938. doi: 10.22271/phyto.2020.v9.i6n.13066

[B72] Singh S. Samal I. Vyas V. Bhoi T. K. Sharma K. Mahanta D. K. . (2025). “ Phytomicrobiome-produced chemosignals: role and implication in plant protection,” in Detection, diagnosis and management of air-borne diseases in agricultural crops ( Springer Nature Singapore, Singapore), 267–291.

[B73] Singh G. Sharma S. (2025). “ Desert ecology and functional aspects of desert ecosystems,” in Textbook of forest science ( Springer Nature Singapore, Singapore), 227–251.

[B74] Singh S. Shukla P. Rajan N. (2021). Traditional utilization & Pharmacological properties of medicinal plants (Karanja). Traditional Utilization Pharmacol. Properties Medicinal Plants 89.

[B75] Singleton V. L. Rossi J. A. (1965). Colorimetry of total phenolics with phosphomolybdic-phosphotungstic acid reagents. Am. J. Enology Viticulture 16, 144–158. doi: 10.5344/ajev.1965.16.3.144

[B76] Somasegaran P. Hoben H. J. (1994). Handbook for Rhizobia: Methods in legume–Rhizobium technology ( Springer Verlag).

[B77] Soumare A. Diedhiou A. G. Thuita M. Hafidi M. Ouhdouch Y. Gopalakrishnan S. . (2020). Exploiting biological nitrogen fixation: a route towards a sustainable agriculture. Plants 9, 1011. doi: 10.3390/plants9081011, PMID: 32796519 PMC7464700

[B78] Talha Bin Yousaf M. Farrakh Nawaz M. Yasin G. Ahmad I. Gul S. Ijaz M. . (2022). Effect of organic amendments in soil on physiological and biochemical attributes of Vachellia nilotica and Dalbergia sissoo under saline stress. Plants 11, 228. doi: 10.3390/plants11020228, PMID: 35050116 PMC8781470

[B79] Thakur P. Kumar P. Sharma C. L. Sharma U. Sharma N. Ladon T. (2025). Ameliorating potential effects of natural biological formulations and biostimulants on plant health and quality attributes in coriander-fenugreek intercropped strawberry (Fragaria× ananassa Duch.). BMC Plant Biol. 25, 164. doi: 10.1186/s12870-025-06184-8, PMID: 39920644 PMC11804047

[B80] Tilgam J. Sreeshma N. Priyadarshini P. Bhavyasree R. K. Choudhury S. Bharati A. . (2022). “ Rhizosphere engineering for systemic resistance/tolerance to biotic and abiotic stress,” in Re-visiting the rhizosphere eco-system for agricultural sustainability ( Springer Nature Singapore, Singapore), 271–300.

[B81] Tsegaye Z. Gizaw B. Tefera G. Feleke A. Chaniyalew S. Alemu T. . (2019). Isolation and biochemical characterization of Plant Growth Promoting (PGP) bacteria colonizing the rhizosphere of Tef crop during the seedling stage. J. Plant Sci. Phytopathol. 3, 013–027. doi: 10.29328/journal.jpsp.1001027

[B82] Ullah R. Abbas Z. Bilal M. Habib F. Iqbal J. Bashir F. . (2022). Method development and validation for the determination of potassium (K2O) in fertilizer samples by flame photometry technique. J. King Saud University-Science 34, 102070. doi: 10.1016/j.jksus.2022.102070

[B83] Valiullin L. R. Gibadullin A. R. Egorov V. I. Mukhammadiev R. S. Mukhammadiev R. S. Sakhnov V. V. . (2024). Ensuring tree protection, growth and sustainability by microbial isolates. Sustainability 16, 7837. doi: 10.3390/su16177837

[B84] Vashistha H. Kumar P. Kumar S. (2024). PGP attributes of non-rhizobial bacterial endophytes from root nodules of wild leguminous plant Tephrosia purpurea (L) Pers. Environ. Conserv. J. 25, 942–955. doi: 10.36953/ECJ.27592833

[B85] Villar I. Rubio M. C. Calvo-Begueria L. Pérez-Rontomé C. Larrainzar E. Wilson M. T. . (2021). Three classes of hemoglobins are required for optimal vegetative and reproductive growth of Lotus japonicus: genetic and biochemical characterization of LjGlb2-1. J. Exp. Bot. 72, 7778–7791. doi: 10.1093/jxb/erab376, PMID: 34387337 PMC8664582

[B86] Vyas V. Bhoi T. K. Samal I. Singh S. Mahanta D. K. (2024). Revitalizing degraded soils with agroforestry interventions: opportunities, challenges, and future direction. Agroforestry to Combat Global Challenges: Curr. Prospects Future Challenges, 529–549. doi: 10.1007/978-981-99-7282-1_25

[B87] Wiegel J. (1981). Distinction between the Gram reaction and the Gram type of bacteria. Int. J. Systematic Evolutionary Microbiol. 31, 88–88. doi: 10.1099/00207713-31-1-88

[B88] Yeremko L. Czopek K. Staniak M. Marenych M. Hanhur V. (2025). Role of environmental factors in legume-rhizobium symbiosis: A review. Biomolecules 15, 118. doi: 10.3390/biom15010118, PMID: 39858512 PMC11764364

[B89] Yu X. Shen T. Kang X. Cui Y. Chen Q. Shoaib M. . (2021). Long-term phytoremediation using the symbiotic Pongamia pinnata reshaped soil micro-ecological environment. Sci. Total Environ. 774, 145112. doi: 10.1016/j.scitotenv.2021.145112

