# Supplementary material for: Pheno-morphological and biochemical characterization of root nodules and associated root nodulating bacteria from Pongamia pinnata (L.) Pierre in the arid regions of India
Source: Front Plant Sci. 2025 Dec 18;16:1717750. doi: 10.3389/fpls.2025.1717750 (PMC12756030; doi:10.3389/fpls.2025.1717750)
Supplement: Supplementary file 1 [file Table1.docx]

Supplementary table 1. Physico-chemical properties of *Pongamia pinnata* rhizospheric soils collected from different sites of western Rajasthan

| **Site** | **N (Kg/ha)** | **P (Kg/ha)** | **K (Kg/ha)** | **pH** | **EC (dS m⁻¹)** | **OC (%)** | **Texture** |
| --- | --- | --- | --- | --- | --- | --- | --- |
| S-01 | 102.3 ± 3.4 ^d^ | 7.8 ± 0.4 ^cd^ | 128.5 ± 4.2 ^d^ | 8.3 ± 0.1 ^b^ | 1.21 ± 0.04 ^b^ | 0.24 ± 0.01 ^b^ | Sandy |
| S-02 | 110.6 ± 3.1 ^d^ | 6.9 ± 0.3 ^d^ | 119.6 ± 3.8 ^d^ | 8.4 ± 0.1 ^b^ | 1.18 ± 0.03 ^b^ | 0.21 ± 0.01 ^b^ | Sandy |
| S-03 | 125.8 ± 3.7 ^cd^ | 8.2 ± 0.3 ^cd^ | 136.2 ± 4.0 ^cd^ | 8.2 ± 0.1 ^b^ | 1.26 ± 0.04 ^b^ | 0.27 ± 0.02 ^b^ | Sandy |
| S-04 | 132.4 ± 3.6 ^cd^ | 9.1 ± 0.4 ^c^ | 142.5 ± 4.1 ^cd^ | 8.5 ± 0.1 ^b^ | 1.33 ± 0.05 ^b^ | 0.29 ± 0.01 ^b^ | Sandy |
| S-05 | 140.2 ± 3.2 ^cd^ | 10.5 ± 0.5 ^c^ | 155.7 ± 4.3 ^cd^ | 8.6 ± 0.1 ^b^ | 1.39 ± 0.04 ^b^ | 0.31 ± 0.01 ^b^ | Sandy |
| S-06 | 152.8 ± 3.9 ^c^ | 11.2 ± 0.4 ^c^ | 163.3 ± 4.0 ^c^ | 8.7 ± 0.1 ^b^ | 1.42 ± 0.03 ^b^ | 0.33 ± 0.02 ^b^ | Sandy |
| S-07 | 164.1 ± 4.0 ^bc^ | 12.5 ± 0.5 ^bc^ | 170.6 ± 4.2 ^c^ | 8.5 ± 0.1 ^b^ | 1.45 ± 0.05 ^b^ | 0.35 ± 0.01 ^b^ | Sandy |
| S-08 | 172.6 ± 3.8 ^bc^ | 13.1 ± 0.4 ^bc^ | 178.4 ± 4.1 ^bc^ | 8.6 ± 0.1 ^b^ | 1.47 ± 0.04 ^b^ | 0.36 ± 0.02 ^b^ | Sandy |
| S-09 | 180.2 ± 4.1 ^bc^ | 13.7 ± 0.3 ^bc^ | 185.6 ± 4.3 ^bc^ | 8.7 ± 0.1 ^b^ | 1.52 ± 0.04 ^b^ | 0.37 ± 0.01 ^b^ | Sandy |
| S-10 | 192.7 ± 3.9 ^b^ | 14.2 ± 0.4 ^bc^ | 190.4 ± 4.0 ^bc^ | 8.6 ± 0.1 ^b^ | 1.49 ± 0.03 ^b^ | 0.38 ± 0.01 ^b^ | Sandy |
| S-11 | 205.4 ± 3.8 ^b^ | 15.1 ± 0.5 ^b^ | 196.8 ± 4.1 ^b^ | 8.8 ± 0.1 ^a^ | 1.55 ± 0.04 ^b^ | 0.39 ± 0.02 ^b^ | Sandy |
| S-12 | 212.3 ± 4.0 ^b^ | 16.3 ± 0.4 ^b^ | 205.7 ± 4.2 ^b^ | 8.9 ± 0.1 ^a^ | 1.59 ± 0.05 ^b^ | 0.40 ± 0.02 ^b^ | Sandy |
| S-13 | 224.6 ± 3.9 ^ab^ | 17.2 ± 0.5 ^b^ | 215.6 ± 4.0 ^b^ | 8.7 ± 0.1 ^a^ | 1.62 ± 0.04 ^b^ | 0.42 ± 0.01 ^a^ | Sandy |
| S-14 | 238.5 ± 4.1 ^ab^ | 18.4 ± 0.5 ^ab^ | 225.2 ± 4.1 ^ab^ | 8.8 ± 0.1 ^a^ | 1.68 ± 0.05 ^a^ | 0.44 ± 0.02 ^a^ | Sandy |
| S-15 | 246.8 ± 3.7 ^ab^ | 19.5 ± 0.4 ^ab^ | 233.7 ± 4.2 ^ab^ | 8.9 ± 0.1 ^a^ | 1.70 ± 0.04 ^a^ | 0.46 ± 0.02 ^a^ | Sandy |
| S-16 | 259.4 ± 3.8 ^a^ | 20.7 ± 0.5 ^a^ | 242.6 ± 4.3 ^a^ | 8.9 ± 0.1 ^a^ | 1.75 ± 0.05 ^a^ | 0.48 ± 0.01 ^a^ | Sandy |
| S-17 | 265.2 ± 4.0 ^a^ | 21.1 ± 0.4 ^a^ | 248.1 ± 4.1 ^a^ | 9.0 ± 0.1 ^a^ | 1.78 ± 0.04 ^a^ | 0.49 ± 0.01 ^a^ | Sandy |
| S-18 | 272.3 ± 3.9 ^a^ | 22.0 ± 0.5 ^a^ | 255.3 ± 4.2 ^a^ | 9.0 ± 0.1 ^a^ | 1.82 ± 0.05 ^a^ | 0.50 ± 0.02 ^a^ | Sandy |
| S-19 | 280.7 ± 4.1 ^a^ | 22.5 ± 0.4 ^a^ | 262.4 ± 4.0 ^a^ | 9.1 ± 0.1 ^a^ | 1.85 ± 0.05 ^a^ | 0.52 ± 0.01 ^a^ | Sandy |
| S-20 | 289.5 ± 3.8 ^a^ | 23.2 ± 0.5 ^a^ | 270.6 ± 4.1 ^a^ | 9.1 ± 0.1 ^a^ | 1.89 ± 0.04 ^a^ | 0.53 ± 0.02 ^a^ | Sandy |
| F-value | 58.69 | 10.25 | 114.65 | 41.23 | 25.65 | 31.33 |  |
| P-value | <0.001 | <0.001 | <0.001 | <0.001 | <0.001 | <0.001 |  |
| LSD | 1.25 | 0.08 | 2.24 | 0.04 | 0.001 | 0.001 |  |

LSD= least significant difference, Nitrogen (N, Kg/ha), Phosphorus (P, Kg/ha), Potassium (K, Kg/ha), pH (Hydrogen Ion Concentration), Electrical Conductivity (EC, dS/m), Organic Carbon (OC, %).

Supplementary table 2. Growth parameters of *Pongamia pinnata* seedlings grown in rhizospheric soils from different sites of western Rajasthan

| **Site code** | **Plant Height (cm)** | **Number of Leaves** | **Stem Diameter (mm)** | **Root Length (cm)** |
| --- | --- | --- | --- | --- |
| S-01 | 41.2 ± 1.5 ^d^ | 19.0 ± 0.8 ^e^ | 0.42 ± 0.02 ^e^ | 24.5 ± 1.1 ^e^ |
| S-02 | 43.8 ± 1.4 ^d^ | 20.0 ± 0.9 ^de^ | 0.44 ± 0.02 ^de^ | 25.7 ± 1.0 ^de^ |
| S-03 | 45.1 ± 1.6 ^cd^ | 21.0 ± 0.8 ^de^ | 0.46 ± 0.02 ^de^ | 26.3 ± 1.1 ^de^ |
| S-04 | 47.6 ± 1.3 ^cd^ | 23.0 ± 0.9 ^cd^ | 0.49 ± 0.02 ^d^ | 27.9 ± 1.2 ^de^ |
| S-05 | 49.0 ± 1.5 ^cd^ | 24.0 ± 0.8 ^cd^ | 0.51 ± 0.02 ^cd^ | 28.6 ± 1.0 ^d^ |
| S-06 | 50.3 ± 1.4 ^cd^ | 25.0 ± 0.9 ^cd^ | 0.53 ± 0.02 ^cd^ | 29.8 ± 1.1 ^d^ |
| S-07 | 52.1 ± 1.3 ^c^ | 26.0 ± 0.9 ^cd^ | 0.55 ± 0.02 ^cd^ | 31.0 ± 1.0 ^cd^ |
| S-08 | 53.4 ± 1.5 ^c^ | 27.0 ± 0.8 ^cd^ | 0.57 ± 0.02 ^cd^ | 31.8 ± 1.1 ^cd^ |
| S-09 | 55.7 ± 1.6 ^bc^ | 28.0 ± 0.9 ^bc^ | 0.59 ± 0.02 ^c^ | 33.2 ± 1.2 ^cd^ |
| S-10 | 57.9 ± 1.4 ^bc^ | 29.0 ± 0.8 ^bc^ | 0.61 ± 0.02 ^c^ | 34.5 ± 1.1 ^bc^ |
| S-11 | 59.1 ± 1.5 ^bc^ | 30.0 ± 0.9 ^bc^ | 0.62 ± 0.02 ^c^ | 35.4 ± 1.2 ^bc^ |
| S-12 | 60.7 ± 1.6 ^bc^ | 31.0 ± 0.8 ^bc^ | 0.64 ± 0.02 ^bc^ | 36.8 ± 1.1 ^bc^ |
| S-13 | 62.3 ± 1.4 ^ab^ | 32.0 ± 0.9 ^ab^ | 0.65 ± 0.02 ^bc^ | 37.5 ± 1.0 ^ab^ |
| S-14 | 63.8 ± 1.5 ^ab^ | 33.0 ± 0.8 ^ab^ | 0.67 ± 0.02 ^bc^ | 38.2 ± 1.1 ^ab^ |
| S-15 | 65.2 ± 1.6 ^ab^ | 34.0 ± 0.9 ^ab^ | 0.69 ± 0.02 ^ab^ | 39.4 ± 1.2 ^ab^ |
| S-16 | 66.5 ± 1.4 ^ab^ | 35.0 ± 0.8 ^ab^ | 0.71 ± 0.02 ^ab^ | 40.7 ± 1.1 ^ab^ |
| S-17 | 68.1 ± 1.5 ^ab^ | 36.0 ± 0.9 ^ab^ | 0.73 ± 0.02 ^ab^ | 41.8 ± 1.0 ^ab^ |
| S-18 | 69.7 ± 1.6 ^a^ | 37.0 ± 0.8 ^a^ | 0.74 ± 0.02 ^a^ | 42.5 ± 1.2 ^a^ |
| S-19 | 71.4 ± 1.5 ^a^ | 38.0 ± 0.9 ^a^ | 0.76 ± 0.02 ^a^ | 43.6 ± 1.1 ^a^ |
| S-20 | 72.8 ± 1.4 ^a^ | 39.0 ± 0.8 ^a^ | 0.78 ± 0.02 ^a^ | 44.8 ± 1.0 ^a^ |
| F-value | 120.36 | 95.63 | 42.7 | 131.57 |
| P-value | <0.001 | <0.001 | <0.001 | <0.001 |
| LSD | 0.98 | 0.43 | 0.001 | 0.15 |

LSD= least significant difference

Supplementary table 3. Biochemical characterization of root nodules of *Pongamia pinnata* isolates (PP-01 to PP-20) showing phenol, tannin, FRAP, and total antioxidant content

| **Isolates** | **Phenol (mg/g FW)** | **Tannin (mg/g FW)** | **FRAP (µmol Fe²⁺/g)** | **Total Antioxidant (µg AAE/g)** |
| --- | --- | --- | --- | --- |
| PP-01 | 2.10 ± 0.12 ^b^ | 1.65 ± 0.09 ^b^ | 11.4 ± 0.6 ^b^ | 38.2 ± 1.4 ^b^ |
| PP-02 | 2.95 ± 0.10 ^a^ | 2.34 ± 0.08 ^a^ | 16.2 ± 0.4 ^a^ | 51.3 ± 1.2 ^a^ |
| PP-03 | 2.80 ± 0.11 ^a^ | 2.20 ± 0.07 ^a^ | 15.7 ± 0.5 ^a^ | 49.8 ± 1.5 ^a^ |
| PP-04 | 1.75 ± 0.10 ^b^ | 1.30 ± 0.06 ^b^ | 10.0 ± 0.5 ^b^ | 32.0 ± 1.0 ^b^ |
| PP-05 | 2.40 ± 0.12 ^a^ | 1.95 ± 0.08 ^a^ | 14.5 ± 0.6 ^a^ | 45.0 ± 1.3 ^a^ |
| PP-06 | 2.25 ± 0.10 ^a^ | 1.88 ± 0.07 ^a^ | 13.9 ± 0.4 ^a^ | 43.2 ± 1.4 ^a^ |
| PP-07 | 2.25 ± 0.11 ^b^ | 1.70 ± 0.10 ^b^ | 12.1 ± 0.5 ^b^ | 40.0 ± 1.3 ^b^ |
| PP-08 | 3.15 ± 0.09 ^a^ | 2.42 ± 0.08 ^a^ | 17.3 ± 0.6 ^a^ | 54.5 ± 1.2 ^a^ |
| PP-09 | 2.95 ± 0.10 ^a^ | 2.30 ± 0.07 ^a^ | 16.5 ± 0.5 ^a^ | 52.0 ± 1.5 ^a^ |
| PP-10 | 1.80 ± 0.09 ^b^ | 1.35 ± 0.06 ^b^ | 10.2 ± 0.5 ^b^ | 33.5 ± 1.2 ^b^ |
| PP-11 | 2.55 ± 0.08 ^a^ | 2.00 ± 0.07 ^a^ | 14.8 ± 0.6 ^a^ | 46.8 ± 1.3 ^a^ |
| PP-12 | 2.40 ± 0.10 ^a^ | 1.92 ± 0.06 ^a^ | 13.9 ± 0.4 ^a^ | 44.3 ± 1.4 ^a^ |
| PP-13 | 2.00 ± 0.10 ^b^ | 1.60 ± 0.09 ^b^ | 11.0 ± 0.6 ^b^ | 37.5 ± 1.2 ^b^ |
| PP-14 | 2.85 ± 0.11 ^a^ | 2.20 ± 0.08 ^a^ | 16.0 ± 0.5 ^a^ | 50.5 ± 1.1 ^a^ |
| PP-15 | 2.70 ± 0.09 ^a^ | 2.10 ± 0.07 ^a^ | 15.3 ± 0.4 ^a^ | 48.7 ± 1.3 ^a^ |
| PP-16 | 1.70 ± 0.08 ^b^ | 1.25 ± 0.06 ^b^ | 9.8 ± 0.5 ^b^ | 31.0 ± 1.1 ^b^ |
| PP-17 | 2.35 ± 0.10 ^a^ | 1.88 ± 0.07 ^a^ | 13.8 ± 0.5 ^a^ | 43.0 ± 1.2 ^a^ |
| PP-18 | 2.20 ± 0.09 ^a^ | 1.75 ± 0.06 ^a^ | 13.2 ± 0.4 ^a^ | 41.2 ± 1.3 ^a^ |
| PP-19 | 2.05 ± 0.10 ^b^ | 1.60 ± 0.07 ^b^ | 11.2 ± 0.5 ^b^ | 37.8 ± 1.2 ^b^ |
| PP-20 | 2.80 ± 0.11 ^a^ | 2.25 ± 0.08 ^a^ | 15.8 ± 0.5 ^a^ | 49.5 ± 1.2 ^a^ |
| F-value | 45.65 | 98.54 | 41.25 | 53.67 |
| P-value | <0.001 | <0.001 | <0.001 | <0.001 |
| LSD | 0.09 | 0.04 | 0.1 | 0.87 |

LSD= least significant difference, Ferric Reducing Antioxidant Power (FRAP)

Supplementary table 4: Intrinsic antibiotic resistance of 20 RNB isolates (PP-01 to PP-20) against commonly used antibiotics measured as zone of inhibition (mm)

| **Isolate** | **CTR (mm)** | **GEN (mm)** | **COT (mm)** | **LE (mm)** | **NET (mm)** | **TE (mm)** | **AMC (mm)** | **OF (mm)** | **AK (mm)** | **CB (mm)** | **CIP (mm)** | **CM (mm)** | **K (mm)** | **NIT (mm)** | **S (mm)** |
| --- | --- | --- | --- | --- | --- | --- | --- | --- | --- | --- | --- | --- | --- | --- | --- |
| PP-01 | 15 ± 0.6 ^b^ | 21 ± 0.7 ^a^ | 18 ± 0.5 ^b^ | 40 ± 0.8 ^a^ | 20 ± 0.6 ^b^ | 29 ± 0.7 ^a^ | 17 ± 0.5 ^b^ | 35 ± 0.6 ^a^ | 19 ± 0.5 b | 40 ± 0.7 ^a^ | 40 ± 0.6 ^a^ | 18 ± 0.5 ^b^ | 17 ± 0.6 ^b^ | 22 ± 0.5 ^b^ | 40 ± 0.7 ^a^ |
| PP-02 | 10 ± 0.5 ^c^ | 20 ± 0.6 ^a^ | 40 ± 0.8 ^a^ | 40 ± 0.7 ^a^ | 17 ± 0.5 ^b^ | 31 ± 0.6 ^a^ | 12 ± 0.5 ^c^ | 32 ± 0.6 ^a^ | 22 ± 0.5 ^b^ | 35 ± 0.6 ^a^ | 11 ± 0.5 ^c^ | 21 ± 0.5 ^b^ | 20 ± 0.6 ^b^ | 40 ± 0.7 ^a^ | 34 ± 0.6 ^a^ |
| PP-03 | 12 ± 0.6 ^c^ | 14 ± 0.5 ^b^ | 18 ± 0.6 ^b^ | 40 ± 0.7 ^a^ | 18 ± 0.5 ^b^ | 24 ± 0.5 ^b^ | 14 ± 0.6 ^c^ | 34 ± 0.6 ^a^ | 16 ± 0.5 ^b^ | 12 ± 0.6 ^c^ | 40 ± 0.7 ^a^ | 32 ± 0.5 ^a^ | 11 ± 0.5 ^c^ | 20 ± 0.6 ^b^ | 10 ± 0.5 ^c^ |
| PP-04 | 19 ± 0.6 ^a^ | 20 ± 0.7 ^a^ | 16 ± 0.5 ^b^ | 40 ± 0.8 ^a^ | 25 ± 0.7 ^a^ | 28 ± 0.6 ^a^ | 15 ± 0.5 ^b^ | 38 ± 0.6 ^a^ | 21 ± 0.6 ^a^ | 10 ± 0.5 ^c^ | 40 ± 0.7 ^a^ | 40 ± 0.6 ^a^ | 16 ± 0.5 ^b^ | 14 ± 0.5 ^b^ | 16 ± 0.6 ^b^ |
| PP-05 | 10 ± 0.5 ^c^ | 25 ± 0.7 ^a^ | 16 ± 0.5 ^b^ | 39 ± 0.6 ^a^ | 30 ± 0.7 ^a^ | 25 ± 0.6 ^a^ | 10 ± 0.5 ^c^ | 28 ± 0.6 ^a^ | 34 ± 0.7 ^a^ | 21 ± 0.6 ^a^ | 40 ± 0.7 ^a^ | 18 ± 0.5 ^b^ | 37 ± 0.6 ^a^ | 12 ± 0.5 ^b^ | 24 ± 0.6 ^b^ |
| PP-06 | 13 ± 0.5 ^b^ | 18 ± 0.6 ^a^ | 20 ± 0.7 ^a^ | 40 ± 0.8 ^a^ | 22 ± 0.5 ^b^ | 30 ± 0.6 ^a^ | 15 ± 0.5 ^b^ | 38 ± 0.7 ^a^ | 21 ± 0.6 ^a^ | 18 ± 0.5 ^b^ | 40 ± 0.7 ^a^ | 10 ± 0.5 ^c^ | 20 ± 0.6 ^b^ | 22 ± 0.5 ^b^ | 40 ± 0.7 ^a^ |
| PP-07 | 19 ± 0.7 ^a^ | 20 ± 0.6 ^a^ | 14 ± 0.5 ^b^ | 40 ± 0.8 ^a^ | 28 ± 0.6 ^a^ | 33 ± 0.7 ^a^ | 17 ± 0.6 ^b^ | 36 ± 0.6 ^a^ | 23 ± 0.5 ^a^ | 12 ± 0.5 ^c^ | 40 ± 0.7 ^a^ | 35 ± 0.6 ^a^ | 20 ± 0.5 ^b^ | 14 ± 0.6 ^b^ | 16 ± 0.5 ^b^ |
| PP-08 | 15 ± 0.5 ^b^ | 18 ± 0.6 ^a^ | 40 ± 0.7 ^a^ | 40 ± 0.8 ^a^ | 28 ± 0.6 ^a^ | 30 ± 0.7 ^a^ | 16 ± 0.5 ^b^ | 32 ± 0.6 ^a^ | 21 ± 0.6 ^a^ | 12 ± 0.5 ^c^ | 40 ± 0.7 ^a^ | 40 ± 0.6 ^a^ | 20 ± 0.5 ^b^ | 10 ± 0.5 ^c^ | 16 ± 0.6 ^b^ |
| PP-09 | 18 ± 0.6 ^a^ | 20 ± 0.7 ^a^ | 20 ± 0.6 ^b^ | 40 ± 0.8 ^a^ | 21 ± 0.6 ^b^ | 29 ± 0.7 ^a^ | 15 ± 0.5 ^b^ | 40 ± 0.6 ^a^ | 25 ± 0.6 ^a^ | 12 ± 0.5 ^c^ | 40 ± 0.7 ^a^ | 10 ± 0.5 ^c^ | 18 ± 0.6 ^b^ | 26 ± 0.6 ^a^ | 36 ± 0.7 ^a^ |
| PP-10 | 10 ± 0.5 ^c^ | 14 ± 0.6 ^b^ | 18 ± 0.5 ^b^ | 35 ± 0.6 ^a^ | 20 ± 0.5 ^b^ | 25 ± 0.6 ^a^ | 12 ± 0.5 ^c^ | 28 ± 0.6 ^a^ | 18 ± 0.5 ^b^ | 12 ± 0.5 ^c^ | 34 ± 0.7 ^a^ | 35 ± 0.6 ^a^ | 24 ± 0.5 ^b^ | 14 ± 0.5 ^b^ | 24 ± 0.6 ^b^ |
| PP-11 | 17 ± 0.6 ^b^ | 40 ± 0.8 ^a^ | 40 ± 0.7 ^a^ | 40 ± 0.8 ^a^ | 10 ± 0.5 ^c^ | 20 ± 0.6 ^b^ | 15 ± 0.5 ^b^ | 39 ± 0.7 ^a^ | 40 ± 0.6 ^a^ | 18 ± 0.5 ^b^ | 12 ± 0.5 ^c^ | 35 ± 0.6 ^a^ | 14 ± 0.5 ^b^ | 18 ± 0.5 ^b^ | 40 ± 0.7 ^a^ |
| PP-12 | 12 ± 0.5 ^b^ | 18 ± 0.6 ^a^ | 40 ± 0.7 ^a^ | 20 ± 0.5 ^b^ | 14 ± 0.6 ^b^ | 19 ± 0.5 ^b^ | 15 ± 0.6 ^b^ | 20 ± 0.5 ^b^ | 40 ± 0.7 ^a^ | 10 ± 0.5 ^c^ | 35 ± 0.6 ^a^ | 40 ± 0.7 ^a^ | 24 ± 0.5 ^b^ | 10 ± 0.5 ^c^ | 38 ± 0.7 ^a^ |
| PP-13 | 18 ± 0.6 ^a^ | 20 ± 0.7 ^a^ | 11 ± 0.5 ^c^ | 40 ± 0.8 ^a^ | 20 ± 0.6 ^b^ | 40 ± 0.7 ^a^ | 12 ± 0.5 ^c^ | 25 ± 0.6 ^a^ | 25 ± 0.6 ^a^ | 12 ± 0.5 ^c^ | 21 ± 0.6 ^b^ | 14 ± 0.5 ^b^ | 24 ± 0.6 ^b^ | 40 ± 0.7 ^a^ | 40 ± 0.7 ^a^ |
| PP-14 | 15 ± 0.5 ^b^ | 18 ± 0.6 ^a^ | 20 ± 0.7 ^b^ | 40 ± 0.8 ^a^ | 22 ± 0.5 ^b^ | 30 ± 0.6 ^a^ | 18 ± 0.5 ^b^ | 38 ± 0.6 ^a^ | 20 ± 0.5 ^b^ | 12 ± 0.5 ^c^ | 40 ± 0.7 ^a^ | 35 ± 0.6 ^a^ | 16 ± 0.5 ^b^ | 18 ± 0.5 ^b^ | 24 ± 0.6 ^b^ |
| PP-15 | 10 ± 0.5 ^c^ | 20 ± 0.7 ^a^ | 15 ± 0.5 ^b^ | 39 ± 0.7 ^a^ | 18 ± 0.5 ^b^ | 28 ± 0.6 ^a^ | 14 ± 0.5 ^b^ | 32 ± 0.6 ^a^ | 18 ± 0.5 ^b^ | 10 ± 0.5 ^c^ | 40 ± 0.7 ^a^ | 12 ± 0.5 ^c^ | 21 ± 0.6 ^b^ | 14 ± 0.5 ^b^ | 26 ± 0.6 ^a^ |
| PP-16 | 12 ± 0.6 ^b^ | 18 ± 0.5 ^a^ | 20 ± 0.7 ^b^ | 38 ± 0.7 ^a^ | 22 ± 0.6 ^b^ | 29 ± 0.5 ^a^ | 17 ± 0.5 ^b^ | 35 ± 0.6 ^a^ | 21 ± 0.6 ^a^ | 12 ± 0.5 ^c^ | 40 ± 0.7 ^a^ | 15 ± 0.5 ^b^ | 20 ± 0.5 ^b^ | 18 ± 0.5 ^b^ | 24 ± 0.6 ^b^ |
| PP-17 | 18 ± 0.6 ^a^ | 22 ± 0.7 ^a^ | 18 ± 0.5 ^b^ | 40 ± 0.8 ^a^ | 20 ± 0.6 ^b^ | 40 ± 0.7 ^a^ | 15 ± 0.5 ^b^ | 40 ± 0.6 ^a^ | 25 ± 0.6 ^a^ | 12 ± 0.5 ^c^ | 40 ± 0.7 ^a^ | 20 ± 0.5 ^b^ | 18 ± 0.6 ^b^ | 22 ± 0.5 ^b^ | 40 ± 0.7 ^a^ |
| PP-18 | 15 ± 0.5 ^b^ | 18 ± 0.6 ^a^ | 20 ± 0.7 ^b^ | 40 ± 0.8 ^a^ | 22 ± 0.5 ^b^ | 28 ± 0.6 ^a^ | 17 ± 0.5 ^b^ | 36 ± 0.6 ^a^ | 21 ± 0.6 ^a^ | 12 ± 0.5 ^c^ | 35 ± 0.7 ^a^ | 18 ± 0.5 ^b^ | 20 ± 0.6 ^b^ | 14 ± 0.5 ^b^ | 24 ± 0.6 ^b^ |
| PP-19 | 12 ± 0.5 ^c^ | 20 ± 0.7 ^a^ | 18 ± 0.5 ^b^ | 40 ± 0.8 ^a^ | 20 ± 0.6 ^b^ | 29 ± 0.7 ^a^ | 16 ± 0.5 ^b^ | 34 ± 0.6 ^a^ | 22 ± 0.5 ^b^ | 12 ± 0.5 ^c^ | 40 ± 0.7 ^a^ | 15 ± 0.5 ^b^ | 18 ± 0.6 ^b^ | 16 ± 0.5 ^b^ | 26 ± 0.6 ^a^ |
| PP-20 | 15 ± 0.5 ^b^ | 18 ± 0.6 ^a^ | 20 ± 0.7 ^b^ | 40 ± 0.8 ^a^ | 22 ± 0.5 ^b^ | 30 ± 0.6 ^a^ | 18 ± 0.5 ^b^ | 35 ± 0.6 ^a^ | 21 ± 0.6 ^a^ | 12 ± 0.5 ^c^ | 40 ± 0.7 ^a^ | 20 ± 0.5 ^b^ | 21 ± 0.6 ^b^ | 18 ± 0.5 ^b^ | 24 ± 0.6 ^b^ |
| F-value | 68.59 | 110.3 | 45.68 | 98.69 | 44.36 | 25.86 | 102.7 | 36.56 | 94.28 | 52.63 | 111.3 | 129.6 | 75.96 | 90.25 | 64.71 |
| P-value | <0.001 | <0.001 | <0.001 | <0.001 | <0.001 | <0.001 | <0.001 | <0.001 | <0.001 | <0.001 | <0.001 | <0.001 | <0.001 | <0.001 | <0.001 |
| LSD | 0.12 | 0.24 | 0.31 | 0.16 | 0.1 | 0.37 | 0.22 | 0.36 | 0.27 | 0.19 | 0.34 | 0.44 | 0.38 | 0.27 | 0.14 |

LSD= least significant difference, CTR (Ceftriaxone), GEN (Gentamicin), COT (Co-Trimoxazole, a combination of Trimethoprim and Sulfamethoxazole), LE (Levofloxacin), NET (Netilmicin), TE (Tetracycline), AMC (Amoxicillin-Clavulanic acid), OF (Ofloxacin), AK (Amikacin), CB (Carbapenem, a class including Imipenem or Meropenem), CIP (Ciprofloxacin), CM (Chloramphenicol), K (Kanamycin), NIT (Nitrofurantoin), S (Streptomycin)
